# A global overview of anatomical science education and its present and future role in biomedical curricula

**DOI:** 10.1002/ase.70137

**Published:** 2025-10-21

**Authors:** Michael Hortsch, Virginia Claudia Carneiro Girão‐Carmona, Ana Caroline Rocha de Melo Leite, Ilias P. Nikas, Margaret K. Gatumu, Nii Koney‐Kwaku Koney, Benjamin Arko‐Boham, Doris George Yohannan, Aswathy Maria Oommen, Yan Li, Jian Yang, Alexandra F. Trollope, Amanda J. Meyer, Sonya E. Van Nuland

**Affiliations:** ^1^ Department of Cell and Developmental Biology University of Michigan Medical School Ann Arbor Michigan USA; ^2^ Department of Learning Health Sciences University of Michigan Medical School Ann Arbor Michigan USA; ^3^ Department of Morphology Federal University of Ceará Fortaleza Brazil; ^4^ University of International Integration of the Afro‐Brazilian Lusophony Redenção Brazil; ^5^ Medical School University of Cyprus Nicosia Cyprus; ^6^ Brunel Medical School, College of Health, Medicine and Life Sciences Brunel University of London Uxbridge UK; ^7^ Department of Anatomy, University of Ghana Medical School, College of Health Sciences University of Ghana Accra Ghana; ^8^ Department of Anatomy Government Medical College Thiruvananthapuram India; ^9^ Department of Anatomy Government Medical College Idukki India; ^10^ Department of Human Anatomy, Histology, and Embryology Fudan University Shanghai China; ^11^ School of Biomedical Sciences The University of Hong Kong Hong Kong China; ^12^ Discipline of Anatomy and Pathology, College of Medicine and Dentistry James Cook University Townsville Queensland Australia; ^13^ Department of Cell Biology and Anatomy Louisiana State University Health Sciences Center New Orleans Louisiana USA

**Keywords:** basic sciences, cell biology, developmental biology, education, embryology, gross anatomy, histology, neuroanatomy

## Abstract

The four main anatomical sciences, gross anatomy, histology, neuroanatomy, and embryology, are fundamental subjects for most health professionals and biomedical students. Usually taught as part of preclinical basic science training, the anatomical sciences provide a structural understanding of human or animal bodies at both macroscopic and microscopic levels. This overview characterizes how the anatomical sciences are currently taught around the globe, highlighting similarities, differences, and recent curricular transformations that were partially in response to the COVID‐19 pandemic. Globally, educators of the anatomical sciences navigate similar pressures, including expectations of curricular integration and reduced time for anatomical teaching. Student‐centered teaching approaches and e‐learning technologies have been adopted across many regions, transforming how educators engage their learners. However, not all educators are provided with technological resources to facilitate such educational advancements, particularly in regions where economic inequality and poor infrastructure hinder access to the internet. Though ethical standards guiding the procurement of human bodies have evolved over time, the sources of human bodies that academic institutions use for anatomy education vary widely. Specific regional issues complicate many aspects of anatomical science education, challenging educators to adopt novel teaching approaches. Despite some differences, every global region appears to be moving in a similar direction. However, where academic institutions fall on that trajectory differs for specific regions/countries. How these educational and technological changes influence anatomy education should be carefully considered for the strengths and weaknesses they provide and the opportunities and threats they bring.

## Graphical abstract

How the anatomical sciences are taught worldwide is constantly changing. Common themes are a reduction of teaching time and the inclusion of e‐learning and new pedagogical approaches. Although there is a common direction of change, where different global regions place along that trajectory often correlates with their economic status.

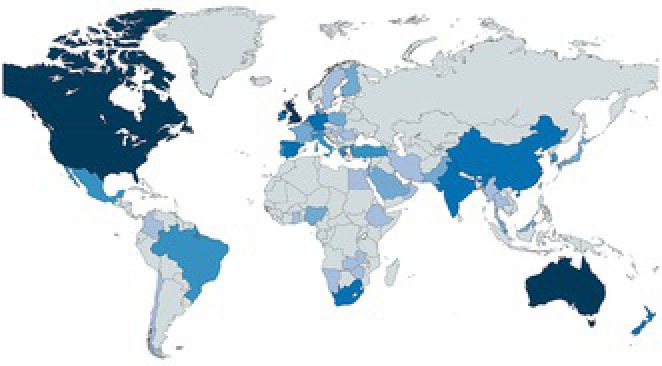

## INTRODUCTION

The anatomical sciences provide an understanding of the structures, functions, and development of biological bodies, organs, and tissues of metazoic species. The word anatomy is derived from the Greek ἀνατέμνειν (ana temnein), meaning “to cut up or to dissect,” which is a central aspect of all anatomical fields. Divided into four distinct major disciplines (gross anatomy/macroscopic anatomy, histology/microanatomy, embryology/developmental biology, and neuroanatomy), the anatomical sciences are keystones of most human and veterinary health sciences and biomedical educational programs, as well as for some non‐scientific professions including medical illustrators and artists. The breadth and depth of anatomical knowledge taught across different professional occupations vary significantly and often depend on required competencies and future job responsibilities. Poor comprehension of the spatial organization of anatomical structures can lead to negative clinical outcomes for patients.^1^, ^2^, ^3^

Traditionally, both gross anatomy and histology have relied on specific modalities to teach learners about the organization and three‐dimensional relationships of biological structures. In gross anatomy, dissection of biological bodies, defined as the act of cutting and separating tissues,^4^ has persisted as the primary teaching method.^5^, ^6^, ^7^, ^8^, ^9^, ^10^, ^11^, ^12^, ^13^, ^14^, ^15^, ^16^, ^17^, ^18^, ^19^ Histology education has predominantly used light microscopy as its central educational technology.^20^, ^21^, ^22^ In contrast, neuroanatomy and embryology use a combination of both dissection and microscopy to educate learners about the structure of the nervous system and developmental processes, respectively.^23^, ^24^ Historically, these approaches and technologies have enabled scientists to describe the structure of metazoan biological systems, fostering a better understanding of the underlying physiological processes and functions and of pathological developments.

**FIGURE 1:**
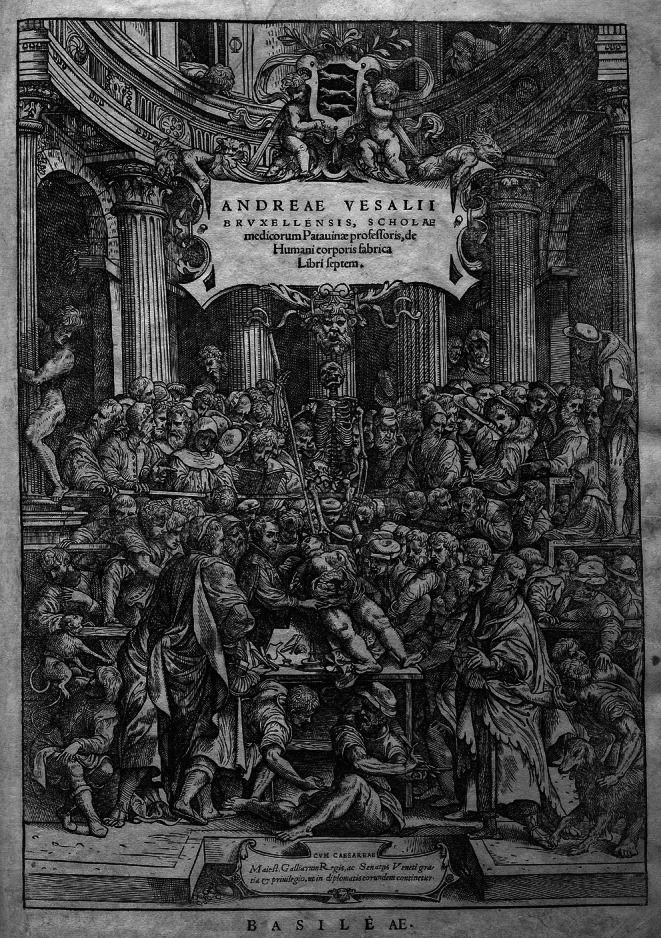
Frontispiece of Andreas Vesalius [1514–1564] “De Humani Corporis Fabrica” (in seven volumes), 1543 Basel, Switzerland.^25^ The image is attributed to the artist Jan van Calcar [ca. 1499–1546] and is in the public domain (courtesy of Wikipedia). The image shows Andreas Vesalius lecturing to a crowd of students and onlookers while performing a public dissection of an executed woman. Several images in the book's volumes are believed to be derived from this dissection.^25^, ^26^

The modern scientific analysis and description of the human body at the macroscopic level started almost 500 years ago with Andreas van Wezel aka Vesalius [1514–1564] (Figure 1).^4^, ^25^, ^26^, ^27^ From the beginning, the anatomical practice of dissection has created ethical, cultural, legal, and religious dilemmas.^6^, ^28^, ^29^, ^30^, ^31^, ^32^ For example, Vesalius obtained the bodies of executed individuals for dissection, a practice, when viewed through a modern lens, must be considered unethical (Figure 1).^31^ In Europe, by the 17th and 18th centuries, public dissections were used “not only for educational purposes, but also as a spectacle for the populace, and as a punitive practice” (Figure 1).^31^ ^(p385)^ Practices of commercializing the human body through public dissections or exhibition, or by selling body parts for profit, have resurged in recent decades.^33^, ^34^, ^35^, ^36^, ^37^ Ethical standards for the recruitment of body and tissue donors for use in anatomical education and research differ widely by geographical region and have evolved over time. However, many academic societies and individuals have lobbied for common ethical standards that support education while protecting the interests and dignity of human body donors.^38^, ^39^, ^40^, ^41^, ^42^, ^43^, ^44^

The scientific description and analysis of living organisms at the microscopic level began nearly 100 years after Vesalius' death with the advent of compound microscopes.^45^ In 1665, using a primitive light microscope, Robert Hooke [1635–1703] coined the term “cell” and published a first observational description of biological cells.^46^, ^47^ However, it took until the first half of the 19th century for biological cells to be identified as the fundamental unit of life on earth.^48^, ^49^, ^50^, ^51^ By the mid‐19th century, the microscopic observation of biological preparations became part of medical education at many European universities.^52^ Since that time, histology or microanatomy usually followed a two‐step model of instruction, a lecture‐style delivery of cellular structure information that is followed by a laboratory component usually involving the analysis of glass slide preparations with light microscopes.^21^, ^22^

Contemporary gross anatomy and histology education have continued to use these traditional methods for teaching, but both are being fundamentally changed by the implementation of new technologies. The consensus among anatomy educators and learners is that dissections should remain the primary educational tool for gross anatomy because they support active learning and the comprehension of complex structural relationships.^53^, ^54^, ^55^, ^56^, ^57^, ^58^ It also emphasizes shared responsibilities and teamwork and plays a role in the development of healthcare professionalism.^8^, ^59^, ^60^, ^61^ However, reduced curricular time has led anatomy educators to decrease the amount of dissection time and to adopt alternative instructional methods.^8^, ^62^, ^63^ These alternatives include the use of wet prosections (predissected bodies),^8^, ^63^ plastinated (predissected and specially preserved dry preparations) or 3D printed specimens,^64^, ^65^, ^66^, ^67^, ^68^, ^69^, ^70^ plastic anatomical models,^71^, ^72^ electronic tables,^73^, ^74^, ^75^, ^76^ virtual and augmented reality approaches,^62^, ^77^, ^78^, ^79^, ^80^, ^81^, ^82^ anatomical websites, videos, and other e‐learning tools.^83^, ^84^, ^85^, ^86^

Histology laboratory education has undergone similar fundamental changes in response to curricular and technological developments. Since 2000, there has been a shift toward viewing web‐based virtual microscopy images (VM) on computer networks rather than using light microscopes and glass slides,^21^, ^87^, ^88^, ^89^, ^90^ Without question, over the recent past, computing technologies have “transformed the practices of anatomical sciences education and research”,^91^ ^(p583)^ promoting a flexible approach to individualized learning at a place, pace, and timing that is controlled by the learner.^92^, ^93^, ^94^ However, as the development and use of modern educational technologies require economic resources that are often only available in affluent countries (mostly in North America, Australia, Aotearoa New Zealand, parts of Europe, and a few other countries), the introduction of these technologies in developing countries often lags behind, resulting in inequities in global anatomy education.^95^, ^96^, ^97^, ^98^

While advancements in education have been heavily influenced by technology, the practices of anatomical sciences education have also seen shifts in pedagogy, learning theories, and changes in student demographics.^99^, ^100^, ^101^ Teaching post‐secondary students has evolved from teacher‐centered didactic instruction to a student‐centered, blended learning approach.^7^, ^102^, ^103^, ^104^ In classroom and laboratory settings, this shift is manifested by the increased use of novel, inquiry‐based educational strategies that are all geared to engage learners.^105^ These include active learning,^106^, ^107^, ^108^, ^109^, ^110^ flipped classroom courses,^111^, ^112^, ^113^ team, problem‐, and case‐based learning,^114^, ^115^, ^116^ and gamification.^117^, ^118^, ^119^, ^120^, ^121^, ^122^, ^123^, ^124^, ^125^, ^126^, ^127^, ^128^

In some countries, medical accrediting bodies have developed competency‐based undergraduate curricula to standardize medical education and ensure curricular enhancement.^129^, ^130^, ^131^, ^132^ Many of these accrediting bodies encourage integrating courses and content to achieve curricular goals, but the “…definitions, interpretations and implementation strategies [vary] greatly among medical schools”.^133^ ^(p785)^ The impetus driving most integration strategies has been to enhance critical thinking, problem‐solving abilities, and the development of clinical reasoning skills.^133^, ^134^ These curricular modifications have fostered the development of core syllabi for the four anatomical sciences, outlining the required knowledge and skills in the context of different professional pathways and responsibilities.^135^, ^136^, ^137^, ^138^, ^139^, ^140^, ^141^, ^142^

The anatomical sciences are foundational and are taught worldwide to most biomedical, medical, and health professional students. Though numerous reports about curricula, educational practices, and educational research have been published at institutional, national, and regional levels, a global level literature overview is missing. The overview below provides a snapshot of the current status of gross anatomy, histology, neuroanatomy, and embryology education based on distinct geographical regions and identifies the internal and external factors that impact anatomical sciences education as strengths, weaknesses, opportunities, and threats in a SWOT framework analysis.^143^

## ANATOMY EDUCATION IN DIFFERENT GLOBAL REGIONS

Each geographical overview below intends to provide the status of anatomical sciences education in different continental or subcontinental regions. Sections include North and South America, Europe, Africa, South and East Asia, and Oceania and describe where and in what format the anatomical sciences are taught and assessed across different educational programs. When available, additional information such as curricular hours dedicated to the anatomical sciences and local challenges is discussed.

The geographical segments were written by active local anatomy educators. These summaries are based on the currently available literature, including non‐English language publications, and the authors' own experiences teaching the anatomical sciences at their respective academic institutions. The literature searches for the individual geographical regions were unstructured and conducted from June 2024 to January 2025 by individual authors with additional support from the lead author.

### Anatomy education in North America

In North America, the anatomical sciences are central components of the preclinical education at allopathic/osteopathic, dental, and veterinary schools,^10^, ^13^, ^144^, ^145^, ^146^ as well as for other professional degree‐granting programs, like nursing, physical and occupational therapy, and non‐professional undergraduate and graduate programs.^147^, ^148^, ^149^, ^150^, ^151^ At a majority of North American allopathic and osteopathic medical schools, the anatomical sciences are part of an integrated preclinical curriculum,^10^, ^13^, ^15^ while for most other professional (veterinary medicine, dentistry) and non‐professional programs, gross anatomy, histology, and embryology are usually taught as independent courses.^146^, ^149^, ^152^, ^153^

**FIGURE 2:**
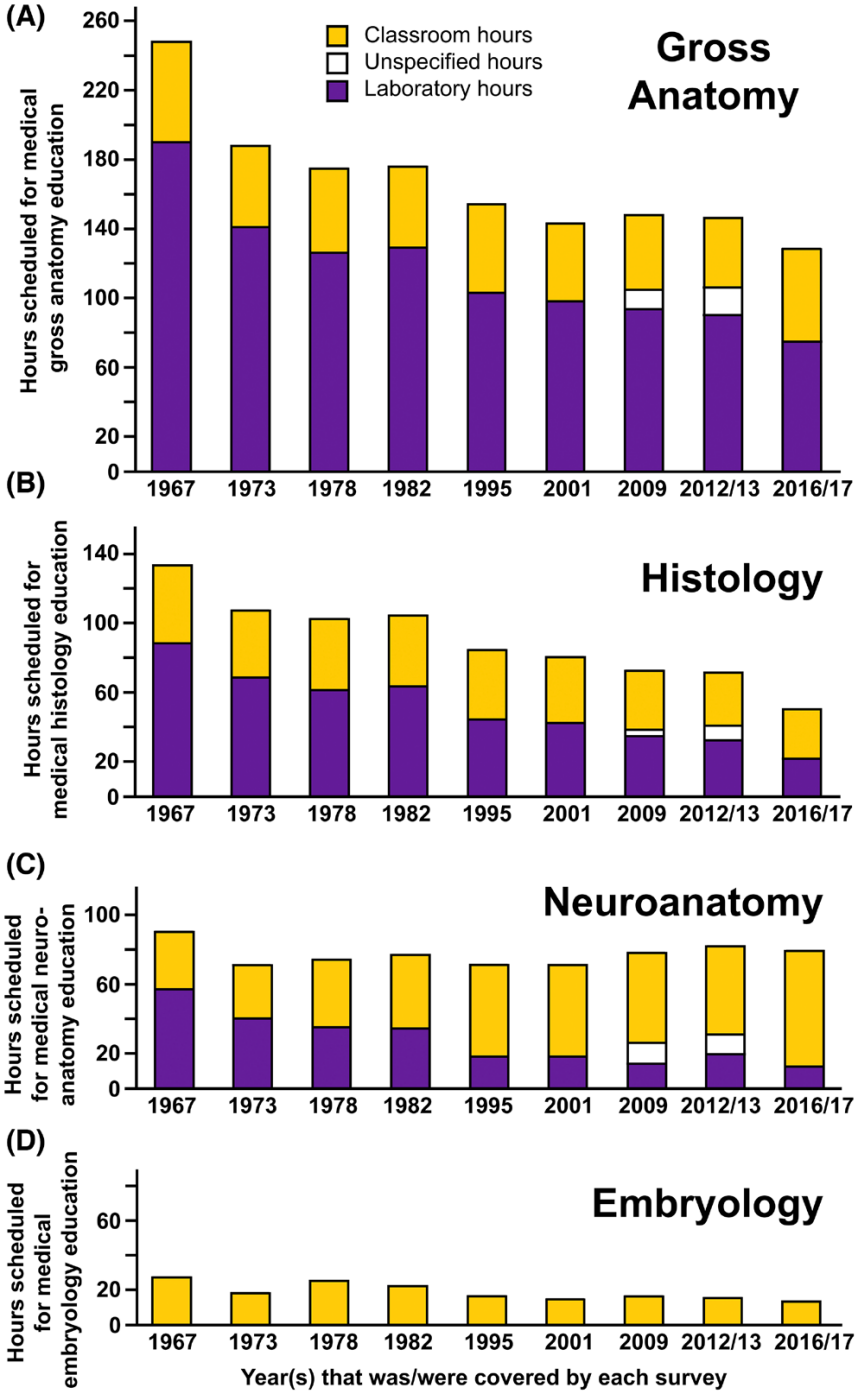
Average time (hours) scheduled in the medical curricula of US allopathic and osteopathic schools for gross anatomy (A), histology (B), neuroanatomy (C), and embryology (D) education covering the time from 1967 to 2017. McBride and Drake^10^ only surveyed allopathic schools in the USA. Time allocated for classroom instruction is indicated in yellow and for laboratory learning in purple. Data for the creation of this figure were extracted from four publications^10^, ^154^, ^156^, ^157^ and are based on surveys sampling the status of anatomical science instruction at the indicated years. The number of schools that responded to the individual surveys ranged from 21 to 65.

Curricular reform in North America has led to a significant reduction in contact hours for gross anatomy, embryology, and histology education over the past 60 years, particularly regarding laboratory instruction.^10^, ^13^, ^15^, ^154^, ^155^ Figure 2 shows the average dedicated teaching hours for histology, gross anatomy, and embryology in the medical curricula of US medical schools. It breaks down the contact hours of histology and gross anatomy into the average number of lecture and laboratory hours that were allocated for undergraduate medical students. Across medical curricula in North America, more contact hours were dedicated to gross anatomy than to any other anatomical science (Figure 2).^13^, ^15^ Mexican medical programs, on average, spent significantly more time teaching gross anatomy and histology than US and Canadian medical schools.^10^, ^13^, ^15^ Compared to US medical schools, Canadian medical schools are scheduling less time for teaching gross anatomy, histology, and embryology.^10^, ^13^ Interestingly, students in veterinary medicine programs in the United States spent a comparable amount of time participating in dissection‐based learning experiences as medical and osteopathic students, despite having to learn the anatomy of multiple species.^146^ However, veterinary medicine students have more extensive preclinical training, including radiology, anesthesia, and toxicology, than their medical school counterparts.^146^ Instruction time for embryology in the United States has remained stable over the same time period, with neuroanatomy education flipping from mostly laboratory to mostly classroom instruction (Figure 2). The least amount of time in preclinical medical education at schools in the United States has always been allocated for embryology.

In North America, anatomical science content is mostly communicated through large group classroom instruction or video recordings.^10^, ^13^, ^15^, ^152^, ^153^, ^158^ Gross anatomy and histology lectures are often paired with laboratory experiences in medical, dental, veterinary, and physical therapy programs, while embryology content is delivered didactically with limited laboratory experiences across most of these programs.^10^, ^13^, ^15^, ^146^, ^151^, ^159^, ^160^ Mexican medical schools are an exception to this trend, where embryology laboratory instruction represents an average of 29% of student contact hours in standalone embryology courses.^15^

Gross anatomy education across different professional and non‐professional undergraduate and graduate programs in North America varies as much as the resources used to teach it. As of 2016, laboratories involving prosection, dissection, or a mix of both remained the primary method of teaching gross anatomy at almost all North American medical schools,^10^, ^13^, ^15^ veterinary schools,^146^ and physical and occupational therapy programs.^148^, ^151^, ^153^, ^161^, ^162^ However, student participation in dissection experiences remained rare in nursing and non‐professional undergraduate programs.^62^, ^163^, ^164^, ^165^ Some exceptions were non‐professional undergraduate programs in Canada,^149^, ^166^ as well as nursing programs at Samford University, Alabama, USA^167^ and McGill University, Canada.^168^

There is a vocal movement in North America advocating for the exclusive use of bodies for anatomical education that have been obtained from voluntary body donation programs.^44^, ^169^, ^170^ Until recently, some unclaimed bodies were still used in some states of the United States and in Mexico, a practice that, due to legal and institutional reforms and a change of awareness and attitude toward body donations, is slowly disappearing.^171^, ^172^, ^173^, ^174^, ^175^

**FIGURE 3:**
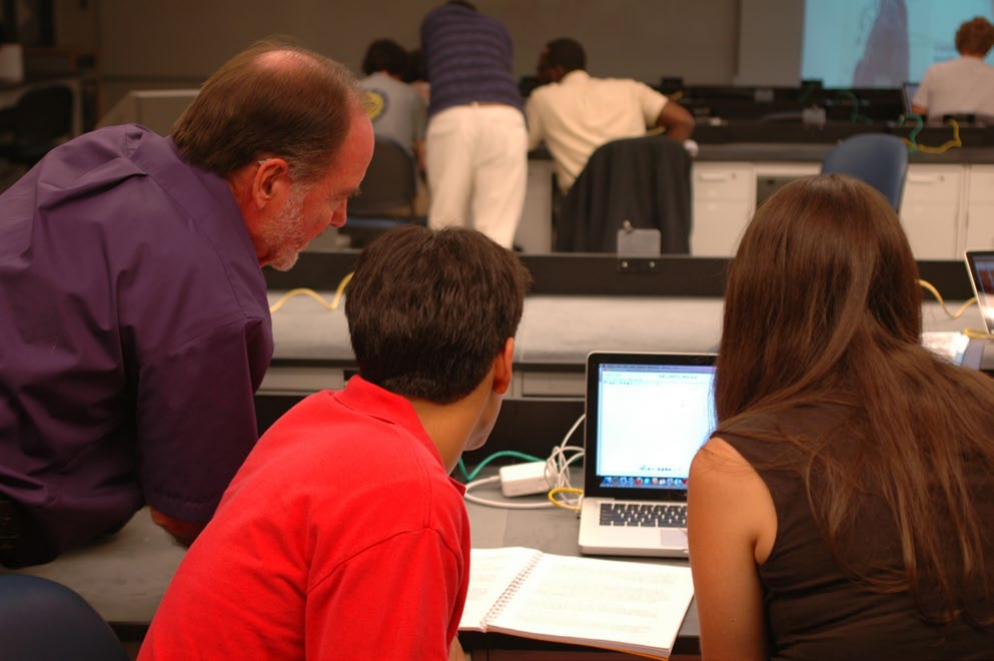
Histology laboratory instruction using virtual microscopy. Dr. Mike Welsh is helping two University of Michigan medical students with the online virtual microscopy Michigan Histology website.^176^ Since this photo was taken in 2010, students at the University of Michigan Medical School and School of Dentistry are no longer offered faculty‐guided laboratory instruction for histology and are expected to work with the website's virtual slides on their own time.^177^, ^178^

Histology education has become increasingly virtual across most programs in North America^10^, ^176^ (Figure 3). Even before the onset of the COVID‐19 pandemic, the introduction of virtual microscopy for histology laboratory education allowed for the development of online histology laboratory sessions.^177^, ^179^, ^180^ Currently, two‐thirds of US medical schools exclusively use virtual histological slides to facilitate laboratory experiences.^10^ However, the use of light microscopy and glass slides has remained the most common histology laboratory activity at Mexican medical schools.^15^

Unsurprisingly, the COVID‐19 pandemic disrupted the use of dissection/prosections across all programs in North America.^163^, ^181^ The reduction in hands‐on dissection‐based learning coincided with a simultaneous increase in the use of anatomy digital resources across most programs in North America,^181^ capitalizing on the growing integration and use of in‐house and commercial anatomical e‐learning tools in North American gross anatomy courses.^91^, ^182^, ^183^

Beyond anatomical e‐learning tools, clinical imaging (x‐ray, ultrasound, magnetic resonance imaging, computer‐assisted tomography) has been increasingly integrated into North American gross anatomy education, particularly in professional undergraduate programs of medicine, dentistry, and the veterinary sciences.^184^, ^185^, ^186^, ^187^ In the last 12 years, clinical imaging has been included in anatomy courses and integrated curricula in 92% of Canadian, 65% of Mexican undergraduate medical programs and in most North American allopathic undergraduate medical programs.^15^, ^188^, ^189^, ^190^ This trend is echoed by 98% of physical therapy programs and 85% of anatomy graduate (M.Sc. and Ph.D.) programs in the United States^151^, ^191^, ^192^, ^193^ and has begun to spread to non‐professional undergraduate programs.^194^ The horizontal and vertical integration of clinical imaging into medical and veterinary undergraduate anatomy courses and vertically through the curriculum in North America was bolstered by curricular reforms focusing on competency‐based medical education and licensing examinations.^10^, ^13^, ^195^, ^196^

Written or online assessments, most often in multiple choice format, are used across medical, veterinary, and dental programs in Canada and the United States to evaluate anatomical sciences knowledge.^10^, ^13^, ^146^, ^197^, ^198^ In most medical and veterinary schools in these countries, gross anatomy knowledge is further assessed through practical examinations that usually require students to identify tagged structures on a clinical image, model, or donor body.^10^, ^13^, ^146^ Unlike the practical assessment of gross anatomy knowledge in medical schools, histology knowledge is often assessed using static images for multiple choice questions, thereby removing the necessity for learners to manipulate the virtual image or the microscope.^197^ Historically, medical schools in Mexico have not been nationally standardized, allowing for autonomy across institutions and departments.^15^ This results in a wide range of assessment practices.

In summary, anatomical education at North American schools has dramatically changed in the past decades. This included the incorporation of modern educational approaches and technologies, the absorption into integrated curricular structures, as well as a considerable reduction of instructional time, especially for the two major anatomical sciences, gross anatomy and histology.

### Anatomy education in South America

**FIGURE 4:**
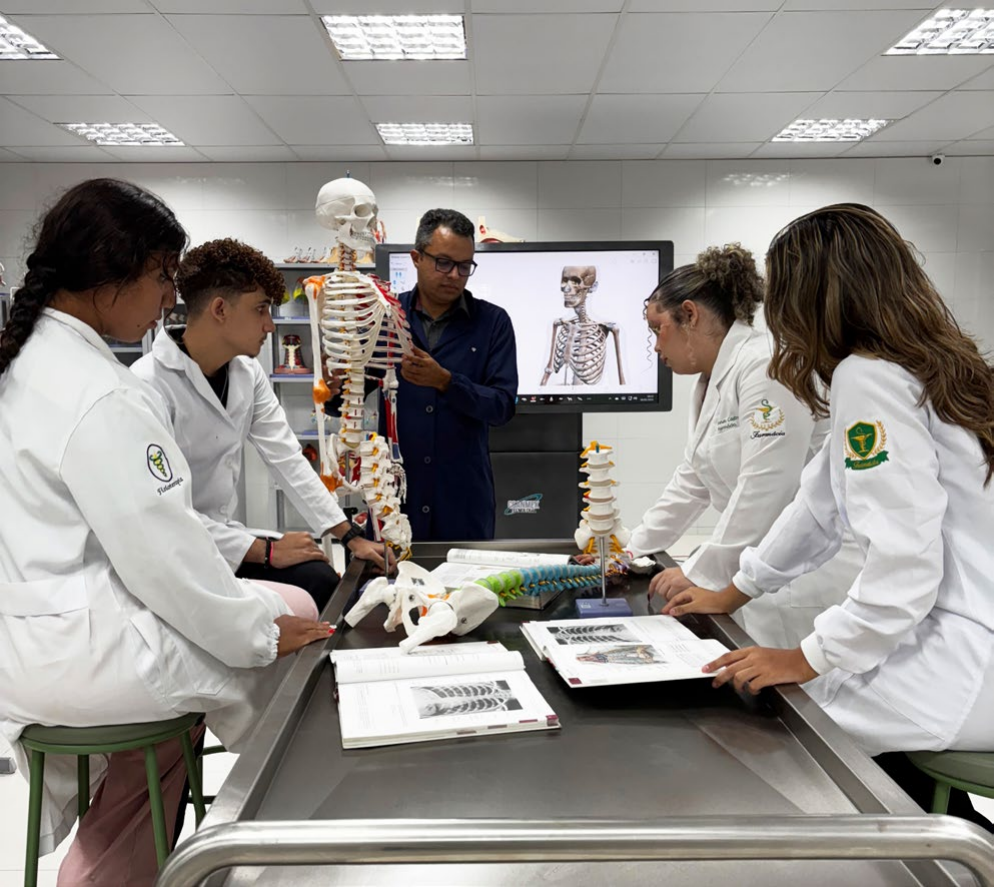
Use of a plastic skeleton model for teaching gross anatomy at a Brazilian university. Dr. Campos teaches the morphology of the axial skeleton to a group of first‐semester graduate students in a Psychology, Physical Therapy, and Pharmacy program at the Fametro University Center (UNIFAMETRO), a private university in Fortaleza, Ceará, Brazil. Plastic bone models are used because the university has no access to ethically sourced human bones for teaching purposes.

In most South American countries, the anatomical sciences are foundational parts of preclinical medical, veterinary, dental, nursing, speech‐language pathology, physiotherapy, pharmaceutical, physical education, biological, and nutritional education programs.^199^, ^200^, ^201^, ^202^, ^203^, ^204^, ^205^, ^206^, ^207^ Anatomy is also taught in ocular optics, optometry, biotechnology, and molecular biology programs and is integrated into the graduate teaching of occupational therapy students, thereby providing a critical framework for the understanding of clinical applications and medical practice.^199^, ^202^, ^208^, ^209^ Across most South American countries, anatomy courses are integrated into medical and surgery curricula.^210^, ^211^ In Brazil, some gross anatomy and histology are taught alongside radiology and pathology or are consolidated into a morphology course.^82^ Some universities in Colombia and Brazil follow a modular, organ‐based approach.^212^ In contrast, in many physiotherapy, nursing, nutrition, and undergraduate biological science programs, gross anatomy, histology, and embryology are still taught as independent courses.^213^ Regardless of the approach used, gross anatomy, histology, and embryology laboratory sessions are independent teaching events at universities across South America.^211^, ^214^ Universities in this geographical region use both traditional and novel teaching strategies and resources for the anatomical sciences, including active learning techniques, team‐based and problem‐based learning approaches, flipped classroom experiences, traditional and e‐textbooks, e‐learning tools, plastic anatomical models, and digital and virtual 3D models (Figure 4).^82^, ^200^, ^212^, ^213^, ^215^, ^216^ In Brazil during the COVID‐19 pandemic, veterinary gross anatomy modules, covering different aspects of animal anatomy, were adapted to accommodate online learning.^206^ Such approaches have fostered a more interactive and autonomous learning environment, which shifts away from the traditional didactic model where the teacher is the primary source of knowledge.^201^ The curricular hours dedicated to the anatomical sciences across different programs and countries/regions in South America are not available from peer‐reviewed publications.

In South America, gross anatomy education has traditionally used a topographic approach in both lectures and dissections. Teaching gross anatomy, particularly neuroanatomy, has been supported through the use of virtual images in atlases and other interactive digital education tools.^206^ In Brazil, medical curricular reform, outlined by the National Curriculum Guidelines, facilitated the integration of clinical imaging technologies into anatomy education.^201^, ^217^ Complementary magnetic resonance images (MRI), ultrasonography (USG) images, computed tomography (CT) scans, and x‐ray films have been integrated into medical gross anatomy education.^199^, ^200^ Consequently, these modalities have become recognized as essential tools in contemporary South American gross anatomy education.^218^

Gross anatomy dissections in most South American medical schools include activities that correlate anatomical knowledge and relationships with clinical applications.^211^ In 2020, all medical educators surveyed at the University of Buenos Aires in Argentina agreed that dissections are fundamental and essential for medical students.^215^ Across many educational institutions in South America, gross anatomy courses are conducted in both large and small group settings.^212^ Small groups typically consist of two to 25 students, with an average of approximately five students per group, which facilitates more focused and interactive learning experiences in the gross anatomy laboratory (Figure 4).^82^, ^199^ In South American veterinary programs, the use of animal cadaveric parts for dissection is often supplemented by plastinated and diaphanized specimens.^202^ The procurement of human bodies for gross anatomy laboratory sessions varies widely within and across different South American countries, with some institutions using only unclaimed bodies and others only donated bodies.^171^, ^210^, ^219^ A movement has been started in South America to establish voluntary body donation programs to ensure a consistent supply of ethically sourced body donors.^210^, ^220^, ^221^

Histology has a long history in South American biomedical education.^222^ At a majority of South American universities, it is part of the first‐year basic science medical/dental curriculum, although some have advocated for its integration into the clinical phase of medical education.^223^, ^224^, ^225^, ^226^ It usually follows the traditional two‐step model, with an initial lecture component that is combined with subsequent laboratory sessions.^90^, ^227^ Modern education approaches to the standard didactic lecture, such as flipped classroom strategies, have been successfully tested.^228^, ^229^, ^230^ With less time being scheduled for histology laboratory sessions, digital microscopy has started to replace traditional light microscopy at some South American universities.^203^, ^223^, ^228^, ^231^, ^232^, ^233^ Other histology e‐learning resources have also been introduced and tried at several South American schools, including social media,^234^ websites,^235^ and digital books.^216^, ^236^ However, the use of virtual microscopy and other digital resources at South American schools is far from universal. It presents challenges for medical education in countries that lack the required technical infrastructure,^227^, ^237^, ^238^ such as personal electronic devices and access to fast and reliable internet, which are needed for equitable educational opportunities.^238^, ^239^, ^240^ Nevertheless, despite these obstacles, the transition to online instruction facilitates the introduction and integration of virtual microscopy and other e‐learning resources, offering new opportunities for enhancing the teaching and learning of histological concepts.^238^, ^241^

Like many institutions around the world, South American universities reported that the COVID‐19 pandemic accelerated a technology shift in anatomy education, including a widespread adoption of online teaching methods.^206^, ^209^, ^242^ Some universities used platforms like Zoom for theoretical and practical classes, incorporated 3D software like the Human Anatomy Atlas,^243^ and live‐streamed laboratory sessions.^244^ However, other institutions lacked the required infrastructure and were unable to adapt their anatomy curriculum to compensate for the loss of in‐person laboratories.^245^ Medical educators surveyed at the University of Buenos Aires in Argentina reported divided opinions about virtual learning; many of these educators felt that students only partially comprehended anatomy when they were taught entirely in a virtual format.^215^ A pre‐COVID‐19 study from Venezuela reported that over 90% of students expressed dissatisfaction with virtual teaching aids, such as anatomy modules, audiovisual media, and radiographs.^246^ Students perceived the e‐learning tools as unrealistic and artificial and argued that such tools should not replace dissections in gross anatomy education.^246^ Though the pandemic accelerated the adoption of e‐learning tools into South American anatomy education, many e‐learning tools, except for textbooks, are only available in English, with limited online resources in Spanish or Portuguese.^216^, ^227^, ^236^, ^247^ In summary, in South America, several considerable challenges remain for the successful implementation of online education for the anatomical sciences.^237^, ^242^

Assessment styles used in gross anatomy and histology have remained stable over time, with the COVID‐19 pandemic representing a temporary shift in format. Prior to the COVID‐19 pandemic, assessments in gross anatomy and histology courses mostly consisted of both theoretical and practical examinations.^248^ During the COVID‐19 pandemic, assessment styles shifted away from practical examinations toward more theoretical examinations.^249^ In Brazil, online modules incorporated quizzes and clinical case studies.^206^ Since the end of pandemic restrictions, practical examinations were reinstated at South American universities, contributing to a significant portion of students' final grade, with quizzes, dissection activities, and clinical correlation workshops making up the rest.^211^, ^249^ Novel assessment methods have been explored in South America, including game‐based examinations, where students identified anatomical and histological structures in a competitive setting.^199^

In summary, an increasing number of South American schools have adopted innovative teaching methods and technologies to improve the quality of gross anatomy and histology education.^202^, ^241^, ^242^, ^249^ The COVID‐19 pandemic accelerated the shift toward online education and blended learning approaches, which was/is a challenge for students from socioeconomic disadvantaged backgrounds.^206^, ^238^ Consequently, there have been calls for curricular reform, advocating for an increased workload, better integration of basic sciences with clinical education, and improved content distribution to enhance anatomical knowledge retention.^201^ As a result of such reforms, there has been a noticeable trend of reducing the time allocated for gross anatomy and histology instruction at South American universities, with traditional dissection and light microscopy being replaced by alternative electronic teaching methods.^213^, ^250^

### Anatomy education in Europe

Among European countries, there is a significant diversity in economic wealth,^251^ as well as educational traditions and systems.^252^ The Bologna Process, which is a voluntary non‐binding intergovernmental agreement started in 1999 and seeks to bring more coherence to higher education systems among European institutions of higher learning.^253^, ^254^ At European universities, the anatomical sciences are essential components of human health science^9^, ^16^, ^255^ and veterinary medicine education.^256^, ^257^ However, the current state of anatomy education is still diverse across European countries. Most of these educational programs start at the undergraduate level after the completion of high (secondary) school.

Across Europe, the bulk of medical curricula integrate gross anatomy teaching with other preclinical disciplines. In Sweden and Spain, the gross anatomy education of physiotherapy and nursing students is integrated with other basic science disciplines.^258^, ^259^, ^260^ In Germany, Spain, Finland, and Italy, medical and dental students experience gross anatomy integrated with clinical imaging, embryology, and histology.^14^, ^261^, ^262^, ^263^, ^264^, ^265^, ^266^ Only two out of 39 surveyed medical schools in the United Kingdom and Ireland had an independent gross anatomy course.^16^ An integration of gross anatomy with clinical disciplines was also reported from other European countries.^12^, ^18^, ^258^, ^259^ In German dental schools, gross anatomy is taught in the preclinical curriculum.^267^ In France, some medical schools offer independent gross anatomy courses.^255^, ^268^, ^269^ Stand‐alone medical gross anatomy courses have also been reported in Denmark,^270^ Croatia,^271^ Poland,^272^ and the United Kingdom.^270^ Some European countries, like Russia, follow a centralized medical curriculum with gross anatomy being first taught by anatomy and later by clinical and surgical departments.^273^

In Europe, synchronous and asynchronous lectures remain the ubiquitous mode for delivering anatomical knowledge to health sciences students.^14^, ^16^, ^18^, ^255^, ^258^, ^259^, ^264^, ^267^, ^271^, ^273^, ^274^, ^275^, ^276^ Lectures are usually complemented by a laboratory component. A survey of 120 gross anatomy teachers from across Europe found that human dissection and/or prosections are still considered the best way to teach gross anatomy.^273^ Consequently, the majority of medical and dental schools in Europe reported the use of human dissection or prosections, which are supplemented by plastic anatomical models and/or digital resources.^9^, ^12^, ^14^, ^16^, ^18^, ^258^, ^259^, ^263^, ^265^, ^273^, ^274^ Only some European medical students do not experience dissections or prosections as part of their gross anatomy education. In Finland and France, the dissection element is optional at some medical schools.^255^, ^264^ In the Netherlands, dissections are offered to a limited number of medical students.^18^ Thirty‐four out of 39 surveyed British and Irish medical schools offer dissection or prosection sessions.^16^ In Turkey, due to an insufficient number of available bodies for gross anatomy education, medical and dental students learn gross anatomy from prosections without a dissection opportunity.^277^ Prosections continue to be used for the gross anatomy education of physiotherapy and nursing students in many European countries.^54^, ^258^, ^259^, ^260^, ^277^, ^278^ European gross anatomy programs exclusively use donated bodies with only a few European countries still relying on unclaimed human bodies.^38^, ^171^, ^277^

The use of digital technologies, like virtual dissection tables, for teaching gross anatomy has become increasingly popular in Europe. Their use alongside dissections is common at Russian medical schools^273^ and complements prosections for medical and physiotherapy students in Sweden.^258^, ^259^ In France, more than half of medical schools surveyed use a virtual dissection table to teach gross anatomy.^255^ Digital visualization resources replacing dissections/prosections have been reported for some medical gross anatomy courses in the United Kingdom^279^ and France.^265^, ^270^ In contrast, the educational value of dissection/prosection as part of veterinary gross anatomy education is widely recognized by veterinary students in several European countries^256^, ^257^, ^280^ and European veterinary programs often coordinate gross anatomy education with histology, physiology, and biochemistry^257^ or with clinical courses.^280^

Histology is taught as part of an integrated medical curriculum at many European universities, especially those in Western Europe.^281^, ^282^ However, there are some European schools where it is a stand‐alone course, specifically programs in the Czech Republic, Romania, Germany, and Greece.^89^, ^281^ This subject remains an indispensable component of various professional courses specifically for medicine, dentistry, veterinary science,^128^, ^283^, ^284^ biology,^285^ biomedical sciences,^241^ physiotherapy,^125^ and sports science.^286^

Like in the United States (Figure 2), curriculum changes and the use of virtual microscopy have resulted in a reduction of teaching hours for histology at some European medical schools.^287^ On average, 24 h are used for histology instruction at medical schools in the United Kingdom and Ireland, which is comparable to the average time dedicated to histology in Canadian medical schools (25 h) but considerably lower when compared to US and Mexican medical school averages (51 h and 125 h, respectively).^10^, ^13^, ^15^, ^16^ However, there are also European universities that dedicate significantly more hours to histology teaching.^281^, ^288^ Teaching hours for embryology have also been reduced in Europe, particularly in integrated curricula.^16^, ^289^, ^290^ This reduction of embryology instruction is not universal in Europe, and some institutions have retained stand‐alone embryology courses.^290^

A traditional two‐step combination of lectures and laboratory sessions is used for histology teaching at most European universities.^89^, ^283^, ^291^, ^292^ Whereas some universities in Europe teach histology in a conventional teacher‐centered mode,^292^ more are implementing a student‐centered approach,^119^, ^284^ such as a flipped classroom model,^282^, ^284^, ^291^, ^293^ gamification strategies,^119^, ^125^, ^126^, ^128^ the inclusion of clinical cases,^281^, ^291^ and/or team‐based learning.^284^ At a few European schools, drawings of histological observations or student‐led presentations are used for deeper learning experiences.^294^, ^295^ Like in other affluent countries, most European medical, dental, veterinary, and biomedical sciences programs rely almost exclusively on virtual microscopy to facilitate histology laboratory instruction.^119^, ^233^, ^282^, ^286^, ^287^, ^296^ However, some institutions still use traditional light microscopy with glass slides for laboratories or follow a blended approach.^281^, ^283^, ^291^, ^294^ In contrast to most medical programs, histology laboratory sessions at the Bachelor of Science degree level often provide hands‐on experiences like slide preparation techniques and the use of histological equipment.^285^

A comparison of medical school neuroanatomy curricula from 11 European countries found differences in the time allocated to this subject.^297^ However, at all these schools, neuroanatomy education is integrated with clinical cases, and many schools offer it during the second year of medical studies.

European countries dealt with the COVID‐19 pandemic in different ways. In many countries, human dissections and face‐to‐face teaching were discontinued and changed to online teaching.^12^, ^266^, ^271^, ^276^, ^298^, ^299^ In a few European countries, the use of prosections^265^ and dissection continued, albeit with smaller student group sizes or reductions in teaching time.^265^, ^300^ With the lifting of pandemic restrictions, face‐to‐face dissection experiences have resumed.^276^, ^299^ However, some curricular changes, like recorded lectures that were introduced during the pandemic, were retained.^299^

European institutions employ a variety of assessment formats across the anatomical sciences. In medical curricula, gross anatomy knowledge is often assessed using oral examinations^14^, ^263^, ^266^, ^268^, ^271^, ^300^ and/or written assessments.^12^, ^14^, ^271^, ^300^ Summative histology assessments across different programs are varied; some institutions exclusively use multiple choice type questions,^16^, ^89^, ^287^, ^291^ while others employ essay questions, spot tests, clinical scenarios, and oral examinations.^16^, ^281^, ^296^ Practical gross anatomy assessments across Europe can take many forms and include online tests using photos and slides of prosected specimens,^12^ in‐person tagged plastic anatomical models and donor specimens (spot test),^271^ and objective structured practical examinations (OSPE).^272^ At many European medical schools, for example, in the United Kingdom and Ireland, a combination of written and spot assessments is used for summative assessments, while formative assessments might include oral, written, or practical exercises.^16^, ^301^

In summary, anatomical education in Europe is still rather heterogeneous between countries, institutions, programs, and for different cohorts of students. However, as 49 European countries are currently signatories of the Bologna agreement,^253^, ^254^ the teaching of the anatomical sciences is slowly becoming more standardized across the continent, easing student and faculty mobility between countries.

### Anatomy education in Africa

The African continent is the home of many developing nations, and the structure of their higher education systems is often based on the universities of their former colonial powers.^302^, ^303^, ^304^ Importantly, the development and rebuilding of their organizational structure is frequently impeded by economic constraints.^302^, ^303^ Though some African countries are making efforts to modernize their medical curricula, many still use traditional teaching methods that do not fully incorporate advances in medical education, such as the use of digital resources, 3D models, and clinical imaging to complement dissections.^305^ These inequities in training across the continent mean that students in some parts of Africa may have unmet educational needs when compared to their peers in more developed countries or other regions/countries of Africa. There are some exceptions to the situation described in this segment. For example, most North African countries bordering the Mediterranean Sea can offer a more technologically advanced anatomy education to their students.^306^, ^307^, ^308^, ^309^, ^310^, ^311^ Similarly, anatomy education at South African universities is comparable to that offered by institutions in more developed countries.^312^, ^313^, ^314^

In general, many African medical and health science schools face significant resource limitations, including underfunding, inadequate facilities, low number of faculty members, a lack of modern teaching tools, and, for gross anatomy, an insufficient supply of bodies for dissection/prosections.^98^, ^305^ For gross anatomy education, many African anatomy departments lack an adequate infrastructure of dissection rooms and resources for embalming and preserving human bodies.^315^ Like faculty in other global regions,^316^ African anatomy educators often find themselves torn between focusing on research for achieving promotions and dedicating time for developing their teaching skills.^314^ Moreover, the integration of information and communication technologies into anatomy education is still in its infancy.^317^ Despite these challenges, there has been a growing recognition of the importance of incorporating innovative technologies and learner‐centered teaching for the anatomical sciences in African biomedical curricula.^76^, ^318^, ^319^

Anatomy courses at most African medical schools are typically stand‐alone courses. Over recent years, some African medical schools have adopted a modular, partially integrated course system for basic medical education.^320^, ^321^, ^322^ These courses combine gross anatomy, histology, embryology, and neuroanatomy and are offered to medical and dental students during their preclinical and clinical years. Physiotherapy, occupational therapy, radiotherapy, radiography, dietetics, nursing, pharmacy, and veterinary science students also take anatomy courses as part of their professional programs.^313^, ^323^, ^324^, ^325^, ^326^, ^327^, ^328^ Some African universities also offer bachelor's, master's, or doctoral (Ph.D.) programs in human anatomy.^320^, ^329^ The duration and structure of these degree programs vary, but they generally span several semesters and include theoretical and practical components.

**FIGURE 5:**
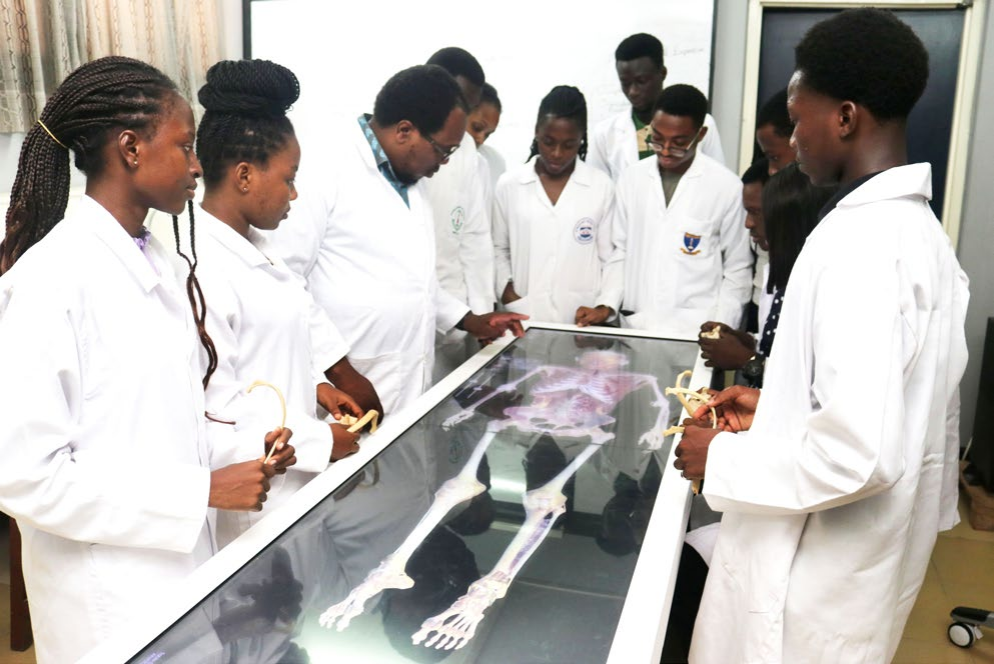
Example of electronic dissection table use to teach gross anatomy in a developing country. The photo shows Dr. Koney instructing a group of University of Ghana physiotherapy and occupational therapy undergraduate students using an Anatomage table (Anatomage Inc., Santa Clara, CA, USA).

Across Africa, traditional teaching approaches remain essential components of gross anatomy education. Didactic lectures accompanied by dissections form the foundation for student learning.^312^, ^330^ Supplemental learning tools such as bone collections, articulated skeletons, and prosected specimens are frequently available.^312^ Many students and faculty across Africa share a strong preference for whole‐body dissection as their primary method of learning.^331^, ^332^, ^333^, ^334^, ^335^, ^336^ Across much of Africa, inadequate infrastructure remains a major barrier to the implementation of computer‐assisted learning.^98^, ^305^ However, e‐learning tools such as virtual dissection tables are being used to supplement gross anatomy education across a small number of institutions (Figure 5).^76^, ^319^, ^337^, ^338^ Similarly, a few medical schools also purchase subscriptions for their learners to access computer programs such as Osmosis and Complete Anatomy (both owned by Elsevier, Amsterdam, The Netherlands).^76^, ^319^, ^339^ Some educators have built in‐house e‐learning tools that incorporate problem‐based^312^, ^340^ and team‐based learning approaches to encourage engagement.^341^

In Africa, the acquisition of bodies for gross anatomy education varies between countries.^342^ Generally, bodies for gross anatomy education are either obtained through willed body donation programs^343^ and/or are unclaimed without consent.^171^ Generally, the reliance on unclaimed bodies is still prevalent due to a lack of well‐established donation programs. In some countries, like Ghana^344^ and Nigeria,^329^ government acts empower the use of unclaimed bodies for dissection at medical schools. The lack of a comprehensive legal framework to facilitate body donations makes it difficult to set up body donation programs that conform with legal and ethical standards and assure the proper handling of donated bodies.^345^ Cultural and religious beliefs may further discourage individuals from donating their bodies. In some African cultures, the body of the deceased is viewed as sacred and should be buried intact, while other communities may have well‐established customs with traditional burial rites to ensure the peaceful transition of the deceased into the afterlife.^345^, ^346^, ^347^ Fear and mistrust are major obstacles to obtaining consenting donors. Further complicating the situation is a low awareness among the countries' general population, as well as their respective policymakers of the need for body donation programs.^344^, ^345^, ^346^, ^347^, ^348^ It should be noted that efforts are being made in several African countries to transition to more ethical practices.^343^, ^344^, ^348^, ^349^, ^350^, ^351^, ^352^

**FIGURE 6:**
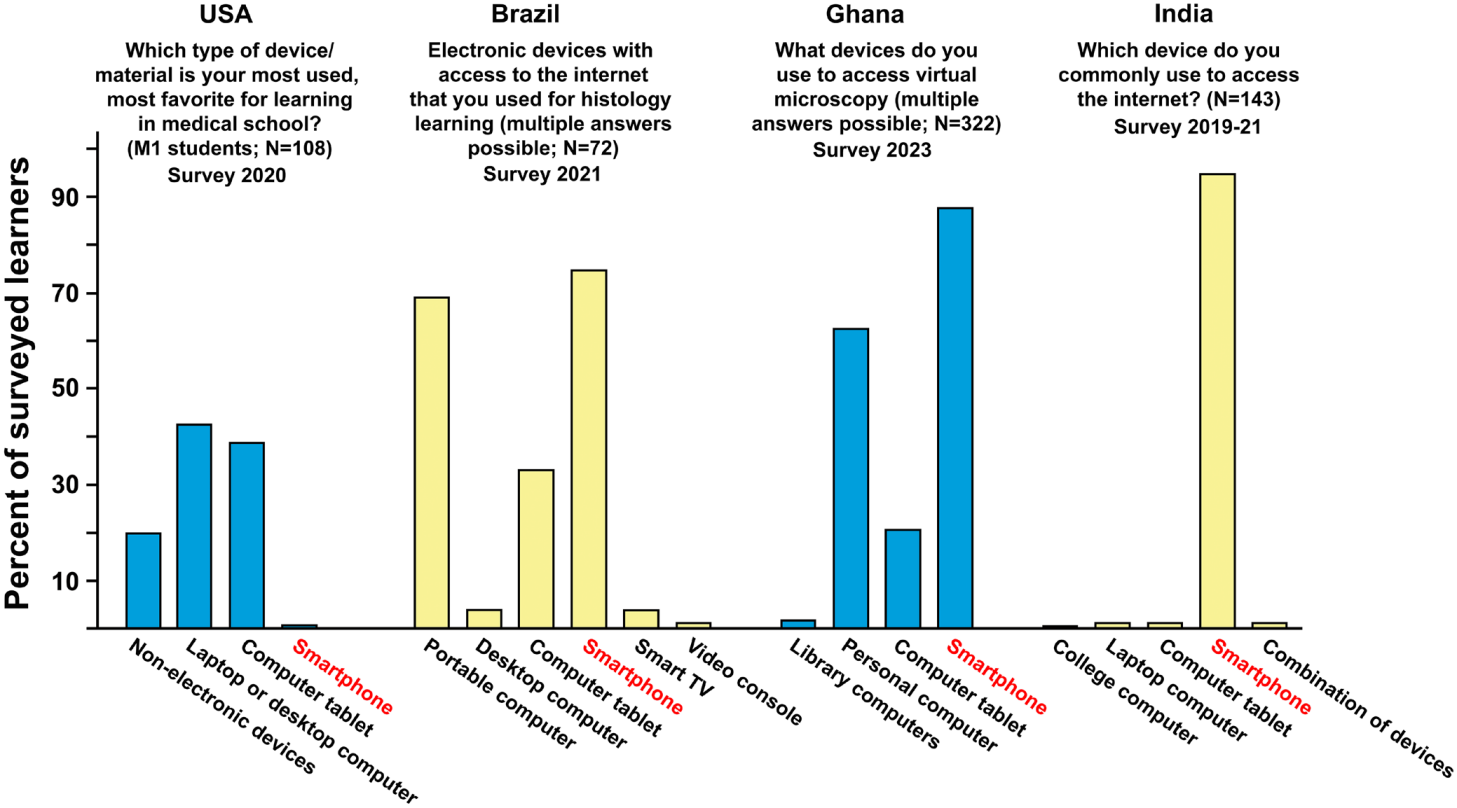
Electronic device usage by students in four different countries. Anatomical science learners from four universities in different countries were asked about their preferred electronic device that they use for studying online. The data from the University of Michigan in Ann Arbor, Michigan, USA, the University of International Integration of the Afro‐Brazilian Lusophony in Redenção, Ceará, Brazil, and Sree Gokulam Medical College in Trivandrum, Kerala, India, were previously published and reformatted for this figure.^238^, ^362^, ^363^ The data from the University of Ghana in Accra, Ghana, are from an unpublished survey of dental and medical students. As the exact wording of the question differed in the four surveys, the question text that was answered by the survey participants is displayed above the relevant bar set. Even though this figure shows data from different surveys that were performed in different years with different groups of students, the results indicate that students in developing countries, where ownership of personal computers is not ubiquitous, find ways to engage in e‐learning by using what is available to them, their smartphones.

Histology education in Africa uses both traditional and modern teaching methods and technologies. Traditional methods are based on light microscope examinations of glass slides with sectioned and dye‐stained tissue samples. The difficulty with traditional teaching tools for histology is that light microscopes and glass slides require expensive upkeep. Without proper maintenance, learners often study with resources that may produce uneven or poor‐quality images. In addition, at many African universities offering traditional histology laboratory sessions, multiple students must share a single light microscope, and broken glass slides diminish students' slide collections. Virtual microscopy platforms offer a modern solution to these problems and are slowly gaining use with African histology teachers and learners.^90^, ^227^, ^353^ They allow students to examine digital histology slides at their own time and chosen location. Given that the creation of in‐house virtual histology slide collections is impractical for most universities in Africa, educators and learners make use of free histology resources that are available on the internet. Noticeably, histology websites that are most often used by African students are exclusively offered by non‐African institutions. Popular examples are the University of California San Diego MedPics,^354^ the University of Michigan Histology website,^176^ Histology Guide,^355^ Histology@Yale,^356^ and other websites. Other e‐learning devices and resources, like podcasts, are also frequently used for histology education in African countries.^357^, ^358^, ^359^, ^360^, ^361^ However, for many African students, access to these sites is problematic, either because of a lack of a personal computing device or internet connectivity.^317^ Most African universities do not provide sufficient computer facilities or support. Therefore, African students often use their smartphones as a learning tool (Figure 6). In contrast, virtual microscopy is routinely used for histology and pathology instruction at Northern and South African universities.^357^, ^364^, ^365^, ^366^, ^367^, ^368^, ^369^ For some time, South Africa has been a leader on the continent for the introduction of modern teaching strategies for histology, like the flipped classroom, gamification, and virtual approaches.^370^, ^371^, ^372^, ^373^

At most African schools, embryology and neuroanatomy are taught as standalone courses or as part of larger teaching modules.^312^, ^374^ Embryology usually involves classroom teaching without a practical component, while neuroanatomy, in addition to lectures, includes laboratory sessions with dissections of the nervous system. To enhance faculty training in neuroscience, the International Brain Research Organization (IBRO, Paris, France) organizes Teaching Tools Workshops (TTW) in Africa and provides training grants.^375^, ^376^, ^377^ These efforts are aimed at providing African educators with the necessary tools to teach neuroscience more effectively.

The COVID‐19 pandemic was a significant turning point for African higher education, leading to a greater adoption of online teaching tools and the use of internet resources.^89^, ^339^, ^378^, ^379^, ^380^, ^381^ In fact, some changes made in response to COVID‐19 have been maintained after pandemic restrictions were lifted.^338^, ^382^ However, the student experience was far from uniform. Due in part to socioeconomic and infrastructural barriers, Nigerian students at one institution reported not being able to attend online sessions.^383^ Students and educators alike struggled to adapt to the new virtual teaching and learning landscape.^384^ If not addressed, the challenges faced by African learners may significantly impede the evolution of anatomy sciences education.^385^

In African medical schools, assessment in the anatomical sciences generally follows a combination of traditional and modern approaches, with variations depending on resources, institutional policies, and curricular structures. Histology and gross anatomy assessments usually have a theoretical and a practical component.^386^ Objective‐type questions (MCQs, matching, short‐answer questions, fill‐in‐blanks, etc.) are increasingly preferred for assessing theoretical knowledge. Laboratory‐based examinations test practical knowledge and competencies involving the identification of structures. They usually have a “steeplechase” (“spotters” or “bell ringers”) format using microscopes, projected or printed micrographs, radiographs, or prosected bodies.

**FIGURE 7:**
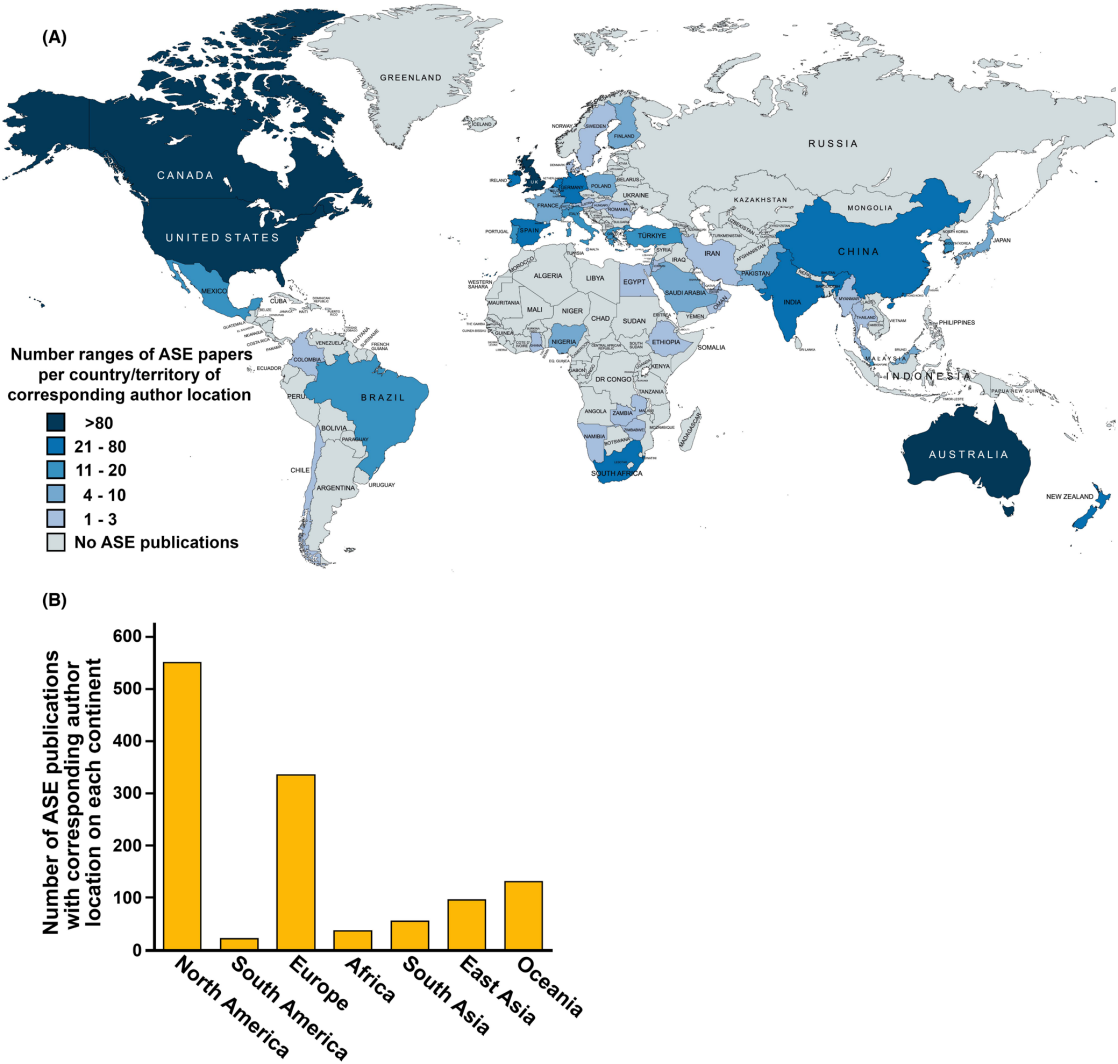
Country and continent affiliations of corresponding authors of Anatomical Sciences Education (ASE) publications. Panel A shows a choropleth map (produced with www.mapchart.net) indicating the 55 different countries/territories of corresponding author locations for 1235 published ASE manuscripts (Volumes 1 to 17). Editorials, errata, and conference abstracts were excluded from this analysis. It should be noted that this figure only provides a very limited snapshot. ASE was selected as it is the only scientific journal that exclusively publishes educational papers for the anatomical sciences. As it is published by the American Association for Anatomy, authors from the United States and Canada are notably overrepresented. A lot of anatomical education work is also published in general medical journals, in anatomical journals that are not specialized in education, or in journals that accept papers on a wide variety of educational topics. Some authors, especially from developing countries, may also choose to submit their work to scientific journals with a more local distribution. In addition, the global movement to open access publication may exclude authors from resource‐limited areas to submit their work to journals with a high open access fee. Panel B shows the number of ASE publications that are associated with corresponding authors from different continents/subcontinents as defined in this article.

The published literature on anatomy education in Africa only captures data from a limited number of higher education institutions and countries. That may bias the view presented in this segment. Despite the presence of many older and recently founded medical schools on the African continent,^387^ there is a noticeable lack of research analyzing anatomy education that has been published in Anatomical Sciences Education and other scientific journals when compared to other continents (Figure 7). In the past, anatomy educators from South Africa have published most of the papers addressing general and specific aspects of African anatomy education (Figure 7).^312^, ^313^, ^314^ However, anatomy educators from other sub‐Saharan countries have started to submit more manuscripts, and the African continent is becoming more visible in the anatomy education literature, some being published in more general education or regional medical journals. This limited research output underscores the need for increased investments in academic research and international collaborations that can enhance the development of anatomy education at African schools. It will also pave the way for introducing innovative teaching methods and technologies, promoting better learning outcomes for African students learning the anatomical sciences.

### Anatomy education in South Asia

South Asian countries not only represent a wide range of population sizes but also differ in their level of economic and technological development and their religious and cultural background.^388^, ^389^ For this summary of anatomical education, India, as the most populous country in the world, will serve as a representative for the South Asian region. The status of anatomy education in India will be compared with that of other South Asian countries. Over the past three decades, like in many other South Asian countries, the number of medical schools in India has grown almost fivefold, making it the country with the highest number of medical schools.^390^, ^391^, ^392^ To improve the quality and consistency of medical degrees across India, modern educational practices have been built around stringent accreditation standards for medical educational institutions, including preclinical anatomy education.^390^ In India, the anatomical sciences are not only part of preclinical training in modern medicine, dentistry, and paramedical professions, but also for alternative medicine approaches such as ayurveda and homeopathy.^393^

Gross anatomy education in India and other South Asian countries relies mainly on dissection^5^ and/or prosections.^17^ In 2020, the National Medical Council of India recommended that virtual dissection tables be added to complement learning in traditional gross anatomy laboratories. In 2023, they revised their recommendations that no more than 10 students should share a body during dissection laboratory sessions.^394^ This caused a severe shortage of bodies across Indian medical, dental, and alternative medical colleges.^395^, ^396^, ^397^, ^398^ The Indian Government and many non‐profit organizations have recently appealed to the public for body donations and have organized body donation drives for academic and research purposes.^200^, ^395^, ^399^ Gross anatomy programs at universities in other South Asian countries are also based on dissections/prosections, often within a problem‐based curriculum.^400^, ^401^, ^402^, ^403^, ^404^

Though intentional body donations have a legal basis in India,^405^, ^406^ unclaimed bodies remain the main source.^171^ Many other Asian countries, including those in the Middle East, use unclaimed bodies, sometimes exclusively.^171^, ^407^ There are a few exceptions to this general reliance on unclaimed bodies, namely Sri Lanka^408^ and Thailand.^409^ This is partially due to different cultural traditions and religious beliefs that are held by individuals in these areas.^171^, ^200^, ^401^, ^410^ The continued use of unclaimed bodies for anatomical sciences education is concerning, particularly in light of the South Asian/Indian bone trade.^36^, ^411^

**FIGURE 8:**
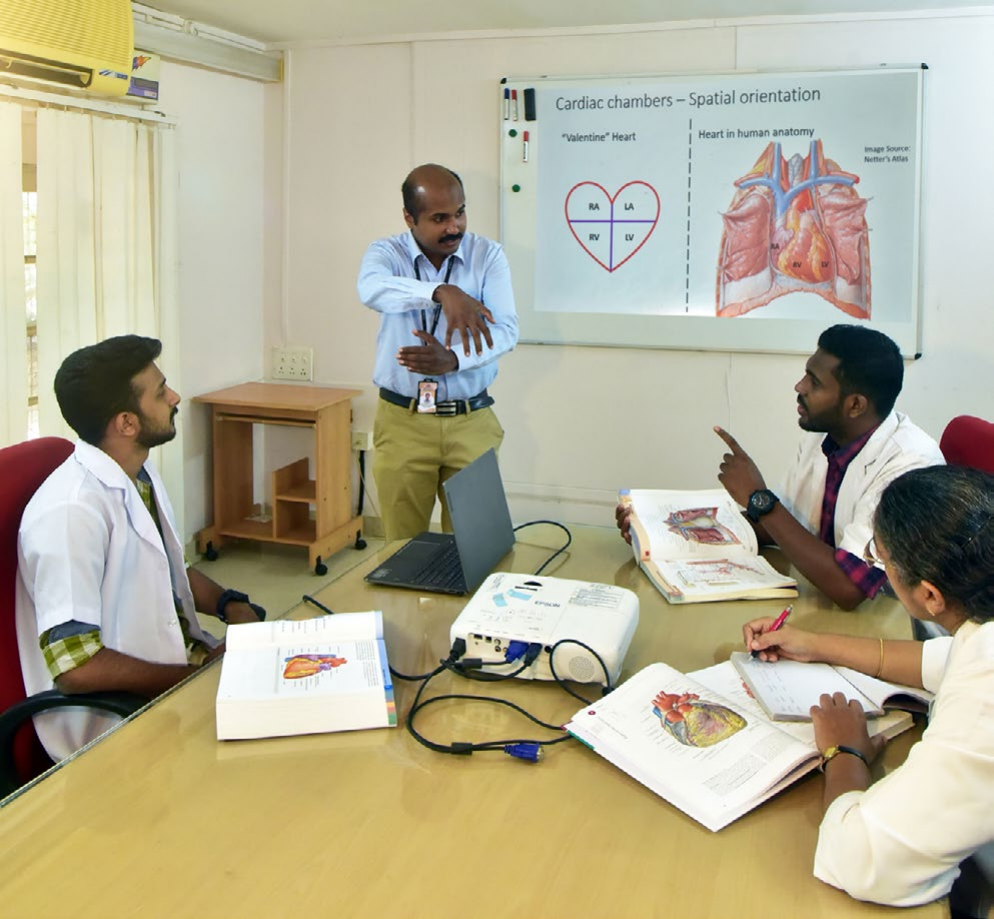
Dr. Yohannan using his “Air Anatomy” technique during a small group session for teaching the spatial orientation of the heart chambers to a class of senior medical students at Government Medical College in Thiruvananthapuram, India.^417^, ^418^ Inspired by traditional Indian theater and dance tradition, he developed this approach to explain complex three‐dimensional anatomical structures using only hand gestures, a technique that can easily be transferred to other resource‐limited teaching environments.

Due to the difficulties of acquiring a sufficient number of bodies for gross anatomy education, some South Asian countries, specifically in the Middle Eastern region, have incorporated a wide range of non‐dissection/prosection teaching strategies.^407^ These include 3D interactive virtual models,^412^ Anatomage tables (Anatomage Inc., Santa Clara, CA, USA),^413^ Sectra tables (Sectra, Linköping, Sweden),^414^ and locally developed 3D stereoscopic visualization techniques.^415^ As reported in some of the above publications, the use of these virtual technologies is sometimes associated with significant increases in students' learning success in gross anatomy and neuroanatomy.^412^, ^415^ However, with few medical schools in the region routinely using these virtual technologies, educational use of these tools lags behind compared to more developed countries.^414^ Surveys of gross anatomy teachers and students in India indicate a preference for a mixed‐method approach, combining dissection/prosection with other e‐learning methods such as virtual, living, and radiological anatomy.^414^, ^416^ This has also resulted in unique low‐technology teaching approaches that are based on the local cultural heritage and can be easily adopted by any anatomy educator (Figure 8).^417^, ^418^, ^419^

The reduction of time allotted to anatomy education has not affected countries like India to the same extent as schools in more developed countries. One reason may be that many medical schools in India still follow a discipline‐based curricular structure.^420^ However, the integration of the basic sciences in medical curricula and early clinical exposure has been increasingly encouraged.^394^ According to the Modified Competency‐Based Medical Education Guidelines of India 2024, the Indian medical curriculum currently allots about 620 teaching hours to the anatomical sciences with an ongoing switch from large group (lecture) teaching toward more small group teaching.^394^ The competencies in the current curricular structure focus on gross anatomy and histology, as well as on neuroanatomy and embryology.^394^ Schools in other South Asian countries, such as in Saudi Arabia, Bahrain, United Arab Emirates, Pakistan, Oman, and Iran, have already moved from a discipline‐based curriculum to a system‐based integrated curriculum, which has reduced the time allotted for practical anatomy instruction.^421^, ^422^, ^423^, ^424^, ^425^, ^426^

The histology component of medical programs in India and other Southeast Asian countries usually covers the histology of cells and tissues, as well as the microscopic structure of most organ systems.^227^ Students in paramedical courses learn histology as a part of their gross anatomy sessions, but with fewer details. The teaching methods used in most of South Asia are similar to those in India.^427^, ^428^ Histology education involves didactic lecture‐style presentations followed by laboratory sessions, usually with stained glass slides and light microscopes. Academic faculty members facilitate students' learning, instructing them on how and what to identify.^227^, ^429^ As the new competency‐based medical education (CBME) curriculum in India stresses the teaching of clinical anatomy, the medical relevance of microscopic structures is now mentioned in the lecture component.^394^ During the laboratory component, emphasis is directed to drawing and labeling microscopic structures, helping students to understand cell and tissue organization. Several published studies suggest that students who draw histology diagrams perform better on related assessments.^371^, ^429^, ^430^, ^431^, ^432^, ^433^ Together with subject knowledge and the ability to identify histological structures, these drawings are also part of student assessments.^429^ Although this traditional version of gross anatomy/histology education is still widespread at Indian colleges, flipped classroom strategies have been launched successfully for both gross anatomy and histology/pathology instruction.^434^, ^435^, ^436^, ^437^

Educational institutions across South Asia face several challenges that impact gross anatomy and histology instruction. Many institutions have experienced considerably higher enrolment rates, difficulties maintaining equipment, such as light microscopes, and a dearth of quality histological slides.^438^ The use of internet resources and computer‐aided instruction, specifically that of virtual microscopy, promises a feasible solution for these challenges.^90^ Though didactic lectures using PowerPoint presentations are the most frequently used method of teaching, other educational resources, like the use of websites, mobile applications, internet‐accessible video demonstrations, self‐directed learning modules, and virtual microscopy, are slowly being integrated into anatomical sciences education at South Asian universities.^97^, ^363^, ^439^, ^440^, ^441^ However, much like countries in Africa and South America, this process has been complicated by outdated technological infrastructures, a lack of student‐owned electronic devices (Figure 6), variable internet access, and reluctance among teaching staff to adopt new educational tools.^97^ Although many schools in the region still use traditional microscopy for histology laboratory instruction, some institutes, like the All India Institute of Medical Sciences, have developed the appropriate infrastructure that is required for e‐learning approaches and the exclusive use of virtual microscopy.^442^, ^443^ Similarly, universities in many Middle Eastern countries regularly use virtual microscopy and other e‐learning resources for histology laboratory instruction.^444^, ^445^, ^446^, ^447^, ^448^

Most biomedical learners at schools in South Asia have embraced new e‐learning technologies, even when their own economic status and limited internet access restrict their use, especially during the COVID‐19 pandemic.^449^, ^450^, ^451^ Like students in other developing countries,^238^, ^452^, ^453^ Indian medical learners quickly adapted by incorporating e‐learning approaches into their education. For many, who lack personal computing devices, their smartphones became their primary learning tool for accessing content (Figure 6).^363^, ^454^, ^455^, ^456^

During gross anatomy and histology assessments at Indian schools, both the students' theoretical knowledge and practical skills are tested. Examinations assess gross anatomy, neuroanatomy, embryology, and histology theoretical knowledge through essays, multiple choice questions, and short answer responses. Practical examinations in gross anatomy include object‐structured practical examinations and discussions using prosections, where students are required to discuss the specimen with the examiner. In histology practical examinations, students are required to identify the slides/structures and substantiate their identification with one or two salient features of the tissue, draw labeled diagrams, and answer questions asked by the examiner. These types of evaluation form part of students' internal assessments and of university examinations.

In summary, many schools in Middle Eastern countries have adopted advanced technologies and educational approaches to teach the anatomical sciences. In contrast, most universities in other areas of Southeastern Asia continue to use a more traditional system of teaching gross anatomy and histology. However, faced with mandated curricular changes, these institutions are slowly converting to new educational methodologies and are introducing modern e‐learning technologies.

### Anatomy education in East Asia

The following segment about anatomy education in Eastern Asia primarily considers the situation in China (mainland China, Hong Kong, and Taiwan), Japan, South Korea, Singapore, Malaysia, and Indonesia. Several of these countries are highly industrialized and affluent, with well‐developed higher education systems.^457^, ^458^ Medical schools in Singapore, Malaysia, and Indonesia are still influenced by their respective colonial pasts and are based on old British, French, or German university systems.^459^ In 2018, mainland China, the largest and most populous country in East Asia, reported 192 universities with clinical medicine majors.^460^, ^461^ Much like other geographical areas, anatomy education in East Asia has undergone significant transformations over the past 20 years, driven by pedagogical innovations, technological advancements, and the impact of the COVID‐19 pandemic.^81^, ^462^, ^463^, ^464^ Anatomy curricula across different East Asian countries and regions exhibit considerable diversity in structure, content, pedagogical approaches, and teaching hours. Even within the same region or country, approaches to curricular designs for the anatomical sciences diverge, as exemplified by the two medical schools in Hong Kong; one has adopted an integrated, learner‐centered teaching approach, while the other maintains a traditional discipline‐based organization.^465^, ^466^, ^467^, ^468^, ^469^

**FIGURE 9:**
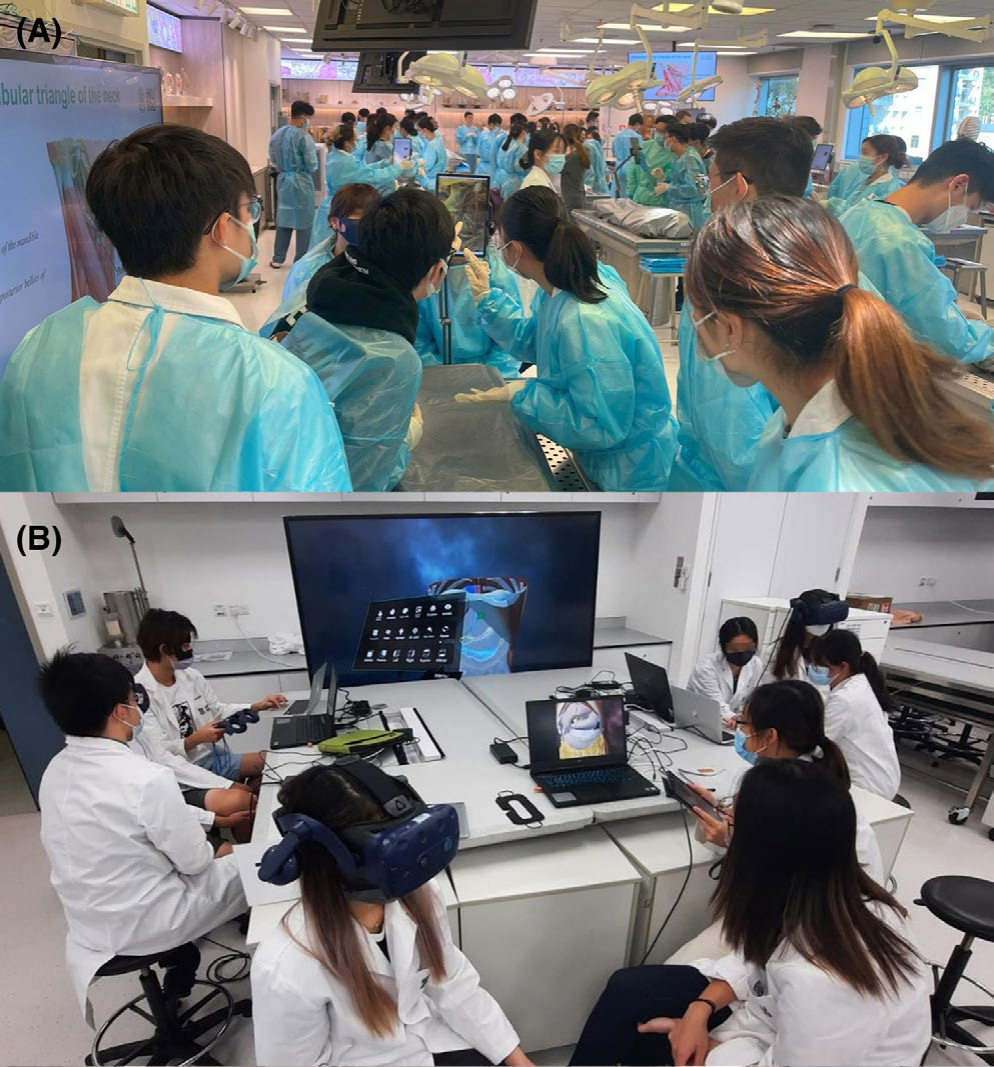
Traditional dissections (Panel A) and virtual reality technologies (Panel B) are used for gross anatomy education at the University of Hong Kong. Photos are courtesy of the Anatomy Education Team of the LKS Faculty of Medicine at the University of Hong Kong.

In mainland China, most medical schools continue to use a traditional dissection‐based approach to gross anatomy. Systemic anatomy is taught in the first and second semesters and regional anatomy in the third, fourth, and fifth semesters.^463^, ^470^, ^471^ However, an 11% reduction in teaching hours over the past 30 years has prompted many medical schools to reform their gross anatomy curriculum, focusing on improving learning efficiency through problem‐based learning and virtual simulations.^470^, ^471^ Curricular modifications have included integrating gross anatomy with other preclinical subjects and emphasizing clinical relevance.^471^ In particular, gross anatomy education in Taiwan separates systems and regional anatomy and combines traditional dissections with modern educational methods.^472^ Recently, virtual and augmented reality technologies have been used to supplement the traditional dissection approach (Figure 9).^473^, ^474^ The National University of Singapore undergraduate medical curriculum uses a modular format, integrating gross anatomy with biochemistry and physiology, while the graduate‐level curriculum employs team‐based learning and multimedia resources.^475^, ^476^, ^477^ These innovations maintained the overall quality of gross anatomy education despite a reduction in teaching hours. In South Korea, gross anatomy curricula have changed by moving to regional gross anatomy teaching and the use of digital tools.^478^, ^479^, ^480^ While face‐to‐face dissection classes have resumed post‐COVID‐19, digital tools and online assessments continue to be widely used. Japanese medical schools have strengthened collaborations between preclinical and clinical departments and incorporated problem‐based learning and digital technologies to create clinically relevant curricula.^81^, ^481^ Analogous changes have been adopted in Indonesian medical schools, where gross anatomy curricula often include prosections, anatomical mannequins, and 3D software.^482^, ^483^ Malaysian universities also utilize similar multimodal approaches and experiential learning to enhance gross anatomy education.^484^, ^485^, ^486^ Much like the rest of the world, the COVID‐19 pandemic accelerated the adoption of new technologies in East Asia, leading to an increased use of digital and virtual modalities/platforms (Figure 9). These advancements are now key components of anatomy education in East Asia, adding to more learner‐centered approaches.^81^, ^467^, ^468^, ^473^, ^474^, ^477^, ^483^

Teaching hours dedicated to anatomy vary significantly across East Asia. However, there is a clear trend toward reducing hours while integrating modern pedagogy and technology. At the University of Hong Kong, medical students receive around 174 h of gross anatomy instruction, including lectures, dissections/prosections, and virtual reality sessions.^466^, ^467^ Korean schools averaged 234.5 h in the 1990s, but there has been a reduction in gross anatomy and basic sciences hours since 2000.^480^ Similar reductions in gross anatomy teaching hours have been reported in mainland China, Singapore, Japan, and other East Asian countries. To support educational quality, innovative pedagogy and technologies have been introduced across many East Asian countries. These learner‐centered methods include the One‐Minute Preceptor model (OMP), digital and virtual reality tools (Figure 9), as well as problem‐based learning.^465^, ^470^, ^471^, ^475^, ^481^, ^487^ In contrast to other East Asian schools and global trends, some medical schools in Taiwan have increased curricular time dedicated to gross anatomy education to accommodate these new technologies. For example, the National Taiwan University College of Medicine allocated 304 h for gross anatomy education, a 72‐h increase compared to the previous curriculum.^472^

Dissection‐ and prosection‐based gross anatomy education remains central at many East Asian schools. Prosections are more commonly used in health‐related programs, like the biomedical sciences, dentistry, nursing, pharmacy, and physiotherapy, where gross anatomy education is less detailed and shorter in duration when compared to medical programs.^43^, ^473^, ^478^, ^488^ Hong Kong, Taiwan, Korea, and Japan schools focus heavily on dissections, which are supplemented by prosections and digital technologies (Figure 9).^81^, ^467^, ^473^, ^474^, ^480^, ^489^, ^490^ Schools in mainland China also emphasize dissections, though instruction hours have decreased.^470^, ^471^ Due to a scarcity of donated bodies, universities in Singapore rely more on prosections and integrate gross anatomy education with clinical learning and digital technologies.^475^, ^476^

Body donation programs have become increasingly important, especially in Hong Kong and Taiwan, where they are integrated into medical humanity training and serve as an important platform for public education.^491^, ^492^, ^493^ Despite the development of these programs, the number of donated bodies is often insufficient, and unclaimed bodies continue to be used at some mainland Chinese, Korean, and Singaporean schools.^470^, ^471^, ^475^, ^479^ Developing body donation programs and addressing cultural and ethical issues around body donation will be crucial for sustaining ethical dissection‐based gross anatomy education.^408^, ^494^, ^495^, ^496^, ^497^, ^498^ Though dissection and prosection remain the cornerstone of gross anatomy education in East Asia, technologies like virtual reality (Figure 9), interactive platforms, digital gross anatomy applications, digital dissection tables, and 3D‐printed models are being increasingly adopted, helping learners to visualize complex gross anatomical structures.^466^, ^467^ Especially in Taiwan and South Korea, augmented and virtual reality have become prominent tools in gross anatomy education.^473^, ^474^, ^480^, ^490^

Histology education, like gross anatomy education, differs between East Asian countries and regions. Generally, the histology curriculum at medical schools in East Asia is delivered either as a standalone or as an integrated course.^499^, ^500^, ^501^ Teaching formats also vary, ranging from traditional face‐to‐face to online courses.^227^, ^241^, ^499^, ^500^, ^501^ Although traditional light microscopy with glass slides is still used, virtual microscopy (or a combination of both technologies) is becoming increasingly popular for histology laboratory instruction in mainland China,^502^, ^503^, ^504^ Hong Kong,^467^ Taiwan,^88^, ^505^ South Korea,^506^, ^507^, ^508^ Japan,^509^, ^510^ Singapore,^501^ Malaysia,^241^ Indonesia,^511^, ^512^ and the Philippines.^513^, ^514^ Flipped classroom designs with student‐centered activities have been introduced in histology and pathology courses across several East Asian universities.^499^, ^515^, ^516^, ^517^ Educators have also implemented problem‐ and case‐based learning, international group‐based learning, and the integration of histology with pathological anatomy to engage learners.^241^, ^459^, ^501^, ^518^

Assessment modalities for the anatomical sciences across East Asia vary but appear to share a desire for ensuring a comprehensive evaluation of learner knowledge. Gross anatomy assessments generally include a mixture of written and oral examinations, prosection‐based practical tests, problem‐based evaluations, home assignments, and often involve continuous formative assessments.^471^, ^473^, ^478^, ^519^, ^520^, ^521^ However, unique strategies are often tailored to each country/region's educational needs and the educational context. In histology, summative assessments include written multiple‐choice questions, as well as oral and practical examination components.^241^, ^518^ Chinese medical schools have adopted both formative and summative assessment strategies including short answer questions.^500^, ^515^, ^522^ At the National University of Singapore, summative assessments include an Objective Structured Practical Examination (OSPE) and a written paper.^501^ Assessments at the National Taiwan University include theoretical and practical portions, each with a variety of question formats.^523^

In summary, educational institutions across East Asia encounter similar challenges in teaching the anatomical sciences as other global regions. These include a reduction in instructional time, increased use of modern electronic technologies, and the adoption of active learning strategies. Notably, the number of peer‐reviewed anatomy education publications from Eastern Asia that have been published in Anatomical Sciences Education has recently increased (Figure 7). Anatomy education in East Asia is still evolving to meet the demands of modern healthcare in the region, balancing traditional practices with pedagogical and technological innovations.

### Anatomy education in Oceania

Fourteen countries comprise the geographical region of Oceania, with the largest by population being Australia, Papua New Guinea, and Aotearoa New Zealand.^524^ The gross domestic product per capita is significantly higher in Australia and Aotearoa New Zealand, compared to all other Oceanian countries.^525^ There are 62 (56 public, 6 private) recognized higher education institutions in Oceania^526^ and 48 teach anatomy. Thirty‐nine are located in Australia, eight in Aotearoa New Zealand, and one in Papua New Guinea.^527^ The Australian Medical Council has accredited 23 medical schools in Oceania: 21 in Australia and two in Aotearoa New Zealand.^528^ The anatomical sciences, however, are taught to a much broader audience than just medical students. They are part of the fundamental years of many biomedical science, allied health science, and science programs in the region.

The published literature on anatomy education in Oceania only captures data from a limited number of higher education institutions, and that may bias the view presented herein. Since 2018, the majority of educational research was performed at affluent Australian urban universities, with only one study originating from a rural campus.^529^ Over the past 6 years, published reports about anatomy education, mostly single‐institution studies, came from 49% of universities in Australia and 38% of universities in Aotearoa New Zealand that offer anatomy courses (Figure 7).^530^, ^531^, ^532^, ^533^, ^534^ There were no recent publications about anatomy education from Papua New Guinea. Educators from Monash University in Australia^535^, ^536^, ^537^, ^538^ and from the University of Otago in Aotearoa New Zealand^532^, ^533^, ^534^, ^539^ were leading contributors to the anatomy education literature.

Despite the anatomical sciences contributing to the foundation of medical practice, by 2010, the time dedicated to teaching anatomy at medical schools in Australasia had markedly decreased from approximately 500 h to a median of 174 h.^540^, ^541^ This reduction of scheduled instructional time, coupled with the growing scope of the anatomical sciences, has raised concerns about the potential impact on learning outcomes. A 2015 survey involving 1100 Australian medical students revealed that senior (clinical) students were 48% less confident in their anatomy knowledge compared to junior (preclinical) students.^542^ Moreover, a 2020 survey of the Royal Australian and New Zealand College of Radiologists revealed that 55% of respondents felt that current graduates had an inadequate level of anatomy knowledge.^543^ Further emphasizing this concern, the Australian Commission for Safety and Quality in Health Care commissioned a systematic review which concluded that physicians and midwives had poor knowledge of perineal gross anatomy.^544^ Recent surveys in Australia and Aotearoa New Zealand have demonstrated that medical students received, on average, 46 h of neuroanatomy instruction^545^ and 21 h of histology laboratory instruction.^546^ Currently, there is a lack of published data regarding the time dedicated to embryology or clinical imaging within anatomy education in Oceania. Students, aware that they need more anatomy instruction, have been advocating for more anatomy laboratory time.^547^, ^548^ These findings highlight an urgent need to reassess and enhance anatomy education to ensure that future healthcare professionals are adequately prepared to practice safely.

According to the literature, anatomical sciences are predominantly taught in undergraduate programs, with a smaller number of postgraduate programs. Anatomy education in the region appears to be focused on allied health students,^549^, ^550^, ^551^, ^552^, ^553^, ^554^ with noticeably less literature on biomedical students,^530^, ^555^, ^556^, ^557^ and even fewer on medical students,^535^, ^536^, ^539^ and science students.^534^, ^558^, ^559^ The teaching strategies reported in these studies demonstrate a preference for didactic methods,^534^, ^551^, ^552^, ^554^, ^556^, ^558^, ^559^ compared to a blended approach,^550^ or flipped classroom models.^553^ Most gross anatomy laboratory classes are not integrated with other pre‐clinical sciences and use face‐to‐face teaching methods that involve prosected body donors,^6^ plastic anatomical models, and clinical images. All schools in Australia and Aotearoa New Zealand exclusively use bodies of consenting donors for education.^560^

In contrast, histology laboratory instruction has become largely integrated using both traditional and virtual microscopy.^227^, ^546^ Two publications contain information on neuroanatomy laboratory instruction, with those in medical programs using donor brains, plastic models, and clinical images^545^ and one in a biomedical program using mostly plastic anatomical models and the Anatomage table (Anatomage Inc., Santa Clara, CA, USA).^561^ These studies highlight the diversity of methodologies used to teach the anatomical sciences in Oceania.

The published literature indicates that digital modalities have yet to fundamentally transform the educational landscape of gross anatomy laboratories in Oceania. Large‐format digital gross anatomy tables received mixed reactions, with a significant number of students expressing a dislike for their use in the gross anatomy laboratory.^555^, ^561^ Additionally, the use of virtual reality headsets made 25% of students dizzy,^562^ and only 37% of dental students reported a comfortable experience using the HoloHuman system.^563^ When given a choice, students preferred the computer desktop version over virtual reality headsets.^564^ These findings suggest that students in Australia still prefer learning gross anatomy from human body donors, where anatomical variation can be better appreciated.^565^

In early 2020, the COVID‐19 pandemic triggered a rapid pivot to online anatomy teaching and learning in Australia and Aotearoa New Zealand.^564^ This is reflected by several studies comparing face‐to‐face and online anatomy teaching modalities.^534^, ^566^, ^567^, ^568^ A 2020 Australian study of veterinary students found that second‐year students with prior face‐to‐face anatomy instruction adjusted better to online teaching than first‐year students, who had received minimal face‐to‐face teaching.^568^ In general, independent of in‐person versus online instruction, students often struggle with the transition from high (secondary) school to university learning.^569^ This is often caused by a lack of foundational skills, support systems, emotional intelligence, and inexperience with different teaching formats. It is worth noting that prior to 2020, Australian educators had already experimented with the online teaching of anatomy.^549^, ^550^, ^555^, ^559^, ^570^, ^571^ Several 2018 reports indicate that short interactive videos with worksheets were highly rated by anatomy learners.^550^, ^555^, ^570^ Additionally, students found online quizzes to be beneficial for deepening their knowledge and understanding and improving their satisfaction.^549^

As clinical imaging has solidified its role as a recognized subdiscipline of anatomy, there has been a marked increase in the number of research papers from academics at institutions employing such imaging devices. Specifically, academics have been using clinical imaging in assessments such as e‐portfolios^572^ and case studies.^561^ Interestingly, 75% of surveyed radiologists in Australia and Aotearoa New Zealand felt that radiology‐supported gross anatomy instruction in combination with digital three‐dimensional teaching tools may eventually replace traditional dissection/prosection‐based methods.^543^ Collectively, these findings suggest a transformative shift may be occurring in anatomical education, highlighting the need for further exploration of the use of clinical imaging in anatomy education.

Recent changes in anatomy education have resulted in calls for curricular reform and the development of core curricula for all anatomy subdisciplines, especially at medical schools in Oceania.^545^, ^546^ Allied health schools in this global region already have made significant progress by developing and implementing core musculoskeletal gross anatomy curricula for physical therapy^532^ and chiropractic students.^573^ These developments will provide a clearer framework for competency level expectations and ensure that all students, regardless of their educational background, can strive to meet established standards. Ultimately, this shift will not only reduce uncertainties about learning outcomes, but it will also improve the quality of anatomy education by spending more time on high‐yield and professionally relevant topics.

## DISCUSSION

The descriptions above provide an overview of the status of gross anatomy, histology, neuroanatomy, and embryology education in distinct geographical regions. All geographical areas describe a shift toward (1) pedagogical changes that favor student‐centered learning, and (2) the adoption of electronic technologies to facilitate learning. Yet, the global overview also demonstrates that individual countries and educational institutions are at different stages of implementing student‐centered learning strategies, as well as integrating and supporting the use of electronic technologies in the classroom. Predictably, affluent countries have access to funding and advanced infrastructures to support these developments when compared to global regions where resources are limited.

### A SWOT (Strengths, weaknesses, opportunties, and threats) analysis of anatomical sciences education in present and future biomedical curricula

In this section, a Strengths, Weaknesses, Opportunities and Threats (SWOT) analysis framework has been used to assess the internal factors (i.e., strengths and weaknesses) impacting anatomical sciences education globally, as well as the external factors (i.e., opportunities and threats) that have the potential to impact anatomical sciences education in the future. The goal of this analysis is to categorize and discuss significant factors that determine the progress of anatomical sciences education globally, including existing factors and those that may occur in the future. In this SWOT analysis, a strength is defined as an internal enhancer of competence, a valuable attribute, while a weakness is an internal diminisher of competence or of resources attributing to or necessary for success. An opportunity is defined as an external enhancer of performance that can be leveraged for benefit, and a threat is defined as an external diminisher of performance that has the potential to reduce accomplishments.^143^

### Strengths of anatomical sciences education

Anatomical expertise will always be an essential foundation for the practice of most medical and veterinary professions, as well as for many branches of biomedical research and the arts.^53^, ^100^, ^574^, ^575^, ^576^ Moreover, knowledge of the anatomical sciences is directly correlated with patient well‐being and clinical outcomes, demonstrating that anatomical sciences education is an internal enhancer of medical professional competence.^1^, ^2^, ^3^ Although no major new discoveries are expected at the gross anatomy level, the scientific understanding of human and animal biology at the cellular, molecular, and developmental levels is far from complete. Exemplified by stem cell therapies and novel vaccine strategies, many new treatments for human diseases are based on cellular approaches. In addition, arising diseases and pathogens present a constant challenge to health care systems. Understanding their causes, progression, and symptomology is often impossible without anatomical and cellular insights, and none can be adequately treated without proper knowledge of the anatomical sciences.

Although some basic anatomy knowledge is important for all health care providers, the required depth of this anatomical expertise varies between different biomedical professions and areas of specialization.^1^, ^577^, ^578^ For example, surgeons and physical therapists need a far more detailed anatomical understanding of the human body than psychiatrists.^148^, ^575^, ^579^, ^580^ Analogous differences in expertise can also be identified for histology, which remains centrally relevant for detecting and diagnosing diseases at the microscopic level.^581^, ^582^, ^583^, ^584^ This need for expected learning outcomes and distinct anatomical competencies across different educational fields is presently being addressed by the publication of discipline‐ and subdiscipline‐specific core syllabi for different biomedical professions and for the different anatomical sciences.^136^, ^137^, ^138^, ^139^, ^140^, ^141^, ^142^, ^585^ Core learning objectives help educators and administrators worldwide to develop curricula that ensure the mastery of basic anatomical content. These examples of standard‐setting syllabi guide student learning, clarify teaching goals, and simplify evaluation.^586^ However, as noted in 2016 by Smith et al. on page 16 of their publication, published learning objectives do not represent a definitive list of expected learning outcomes and knowledge for gross anatomy, histology, neuroanatomy, or embryology that are needed for a professional career, but that “[…] the acquisition of post‐graduation knowledge is specialty dependent”.^137^

The rapid adoption of technological innovations is another valuable attribute in anatomical sciences education. For some time, gross anatomy and histology educators have been at the forefront of medical education innovation.^21^, ^99^, ^587^ Not only were they among the first to employ novel, learner‐centered methods of instruction, but they also made groundbreaking use of modern electronic technologies for teaching and learning.^62^, ^78^, ^90^, ^588^ The rapid adoption of these tools has integrated the learner into the education process, transforming medical educators from “speakers on a stage” to facilitators who lead discussions and model scientific and clinical reasoning. As outlined in this article, not all innovations are currently used equally at the global level. However, interactive e‐learning and virtual reality‐type resources are widely embraced by the anatomy teaching community and will most certainly play important roles in the future.^589^, ^590^ In summary, the arguments presented here make a strong case for maintaining, rather than diminishing, the anatomical sciences as a centerpiece of preclinical biomedical education.

### Weaknesses of anatomical sciences education

The heterogeneous use of modern technology and learner‐centered strategies to teach and engage learners in the anatomical sciences is an internal diminisher of professional competence. Often correlated with the economic wealth of a global region/country, sufficient funding, proper infrastructure, and teacher education are necessary for the success of anatomical sciences education globally.^98^, ^591^ The inequity between countries and regions is further reflected in the geographic origin of novel gross anatomy/histology teaching and learning resources, as well as research articles published in anatomy journals, including but not limited to Anatomical Sciences Education (Figure 7).

Globally, there is widespread experimental use of active and learner‐centered pedagogy in all four of the anatomical sciences sub‐disciplines. However, many students still approach learning the anatomical sciences, especially gross anatomy, through rote memorization, a surface approach characterized by the reproduction of information without an attempt to relate content to a larger context.^592^, ^593^, ^594^, ^595^ Case‐ and problem‐based, learner‐centered strategies provide opportunities for students to contextualize information internally and across sub‐disciplines.^110^, ^116^, ^587^, ^596^, ^597^, ^598^, ^599^, ^600^ However, the preceding geographic segments document how these techniques are still underutilized in professional and non‐professional anatomy programs, especially in developing countries.

Additional internal inhibitors of anatomical science learning success are the number and qualification of teaching personnel. For some time, warnings have been voiced that there is a global shortage of anatomy teachers.^601^, ^602^, ^603^, ^604^ In developing continents particularly, new educational institutions are being opened and the number of students requiring anatomy instruction is quickly growing, triggering concern that demand for well‐trained anatomy educators is outpacing the current supply.^605^, ^606^ Further complicating this situation is the departure of experienced medical educators from developing countries to global regions that are more prosperous, usually attracted by higher‐paying jobs.^607^, ^608^ Some institutions have addressed the educator shortage by turning gross anatomy and histology instruction over to surgeons and pathologists and/or to less qualified individuals.^609^, ^610^ This calls for more anatomy graduate programs, workshops, and courses to produce educators who can fill open teaching positions for the anatomical sciences.^611^, ^612^, ^613^

The anatomical sciences also continue to struggle with public relations, specifically how anatomy knowledge and education is viewed by learners, other educators, and by the public.^614^, ^615^, ^616^ Anatomy educators may be well advised to remind their non‐anatomy colleagues that they have more to offer to the biomedical education community than some old skeletons in their closet. Thoughtful messaging and consistent interactions with the public and academic colleagues are needed to communicate the intrinsic value of the anatomical sciences.^615^, ^617^

### Opportunities for anatomical science education

Integration of the anatomical sciences, both horizontally and vertically, can be used to enhance trainee performance by offering the opportunity to practice a wide range of higher level analytical skills and strategies. Therefore, integration into a larger curricular structure can act as an external enhancer of performance in the anatomical sciences that can be leveraged in many professional programs. Some anatomical science topics are complex and challenging for learners, and usually involve hands‐on, active learning tasks that reflect the higher level learning required to achieve mastery.^197^, ^618^ As the anatomical sciences link structure and development of biological organisms with their biological functions, they are ideally positioned to be integrated in a trans‐disciplinary fashion. Breaking down barriers between different basic sciences and those between basic and clinical departments remains an opportunity that should be pursued to improve the quality of health sciences education worldwide.

The meaningful integration of modern e‐learning resources offers another opportunity for anatomical sciences education. The use of modern e‐learning resources is growing globally, particularly given the impact of COVID‐19 on education.^91^, ^619^ Given that many of these resources are accessed by students using their personal mobile devices (Figure 6),^238^, ^363^, ^454^ these e‐learning tools have the potential to act as equalizers across the global landscape, enabling universities in low‐income countries to deliver quality biomedical sciences education. Within the past 5 years, schools in economically weaker global regions have reported purchasing e‐learning tools, like electronic gross anatomy tables,^76^, ^319^, ^337^, ^620^, ^621^ suggesting a shift in funding that will prioritize learner‐centered anatomical science education in many health science curricula. In addition, many anatomical resources are open, internet‐based tools, like websites,^86^, ^176^, ^355^, ^356^ YouTube channels,^419^, ^622^ and anatomical databases.^623^, ^624^ Sharing these tools can benefit the global anatomy teaching and learning communities.^19^, ^625^, ^626^

Intra‐continental and international collaborations are another external enhancer that can contribute to progress in anatomical science education. New programs do not have to start from zero and can directly learn from leaders in the field.^305^, ^627^, ^628^, ^629^ Encouraging educators and developers from different geographical regions to freely share their ideas, resources, and educational strategies presents a great opportunity for promoting global innovation in the teaching of gross anatomy, histology, neuroanatomy, and embryology.

### Threats to anatomical sciences education

Several developments that are detailed in the descriptive segments of this and other articles are external inhibitors of anatomical science education.^610^ The most frequently cited threat is a continued reduction in teaching times. As indicated by Figure 2 for schools in the USA, dedicated time for gross anatomy and histology laboratory sessions is most at risk. Although, as outlined in this review, this development has mostly impacted schools in North America and Europe,^10^, ^13^, ^16^ other regions of the world appear to follow this trend. The impact of reduced teaching time on histology education was demonstrated by Gribbin et al., who reported that a complete loss of scheduled histology laboratory sessions resulted in a significant reduction of students' histology knowledge and skills.^177^ Other studies have shown that, in gross anatomy, a reduction in hands‐on dissection‐based learning may impede learners' abilities to appreciate anatomical variances and complex situations,^55^, ^630^ resulting in compartmentalized knowledge of facts, especially for lower performing students.^149^, ^539^, ^631^, ^632^ This development may at least partially be ameliorated by the introduction of e‐learning resources and new pedagogic strategies that enable learners to study independently without an instructor being present. The recent COVID‐19 pandemic experience supports this hypothesis and indicates that there are more efficient ways of teaching the anatomical sciences.

In professional schools, time dedicated to anatomical science education is also heavily influenced by national accreditation agencies. Their mandates often override local experts and may not always be tailored to the needs of specific scientific or clinical fields.^633^, ^634^ Despite the general agreement among anatomy educators that dissections/prosections should remain a central component of gross anatomy education, a growing number of medical school administrators make the decision to abandon gross anatomy and histology laboratory sessions, often without consulting the educators themselves.^62^, ^177^, ^395^ In addition, religious and ethical restrictions on body donations and the phasing out of using unclaimed bodies can both cause a shortage of bodies for gross anatomy laboratory instruction and may result in fewer schools offering traditional dissection‐based gross anatomy laboratory education.^220^, ^395^, ^635^

Often, gross anatomy e‐learning tools are touted as alternatives to traditional dissection/prosection laboratories. However, electronic representations often show idealized examples of anatomical structures.^72^, ^100^, ^636^, ^637^, ^638^ Though these tools provide opportunities for improving anatomical education, they should not be taken as magical solutions for all the problems encountered by anatomy educators.^91^ They have limitations, and if used inappropriately by students or educators, may impede rather than foster effective learning.^110^, ^639^, ^640^ The perception that e‐learning tools will motivate anatomy learners and will be effective alternatives to traditional physical specimens is often based on subjective opinions rather than objective scientific evidence.^91^, ^183^, ^641^, ^642^ Though e‐learning tools are popular with learners and some teachers, they often lack the tactile and multi‐sensory feedback that is provided by physical specimens.^183^, ^643^, ^644^, ^645^, ^646^ In summary, with educational technologies remaining an expanding part of anatomical sciences education, teachers and students alike must learn how to use them to their advantage rather than having technology dictate the direction and content of their educational mission.

Another consideration that has the potential to hinder the progress of anatomical sciences education is the continued use of unclaimed bodies for the purposes of gross anatomy studies. Ethical limitations and guardrails for using human bodies in gross anatomy education have significantly changed over time.^31^, ^42^ Vesalius's dissections of executed individuals^26^ (Figure 1), and the abuse of living and dead humans under the Nazi regime^647^, ^648^, ^649^ are just two of many examples of unethical scientific research and anatomical education. Though there is a promising global movement toward the ethical procurement of bodies for gross anatomy education, the use of unclaimed bodies is still widespread in many regions of the world.^171^, ^277^, ^343^ Complicating this controversy are historic anatomical collections housed at institutions around the world, many of which still have educational value but contain items of dubious or unknown origin.^34^, ^36^, ^411^, ^650^, ^651^, ^652^ There are often no easy answers as to how to store, use, or dispose of such collections in an ethical way.

Commercialization of human material is another topic of concern that poses an ethical conundrum for anatomy education.^36^, ^411^, ^653^, ^654^ Recent events like von Hagen's “Körperwelten” exhibitions have raised the interest of the general populace but also made anatomy a public spectacle.^64^, ^655^, ^656^ Educational outreach is crucial for fostering interest in the anatomical sciences, but its commercialization remains a complicated psychological and moral minefield.^37^, ^657^

## LIMITATIONS OF THIS ANALYSIS

Although each continental manuscript segment was written by active educators of the anatomical sciences, many of whom have published previously on a variety of educational topics, this overview provides an incomplete current snapshot of how the anatomical sciences are being taught in different parts of the world. Noticeably, published literature concerning neuroanatomy and embryology education is sparse, and as a result, this overview does not provide a rich description of these components from a global perspective. Additionally, this overview mostly highlights professional learners, such as medical and dental students, as the literature describing the anatomy education of non‐medical and non‐dental students (e.g., physiotherapy, nursing, veterinary, art) is underrepresented.

As this report is not based on actual survey data but rather on the published literature, it does not represent all global regions evenly. Some parts of the world, often economically weaker regions, are underrepresented in the scientific literature, specifically in Anatomical Sciences Education (Figure 7) and, consequently, also in this overview. In addition, with educational realities constantly changing, the situation in the published literature may no longer adequately reflect current conditions. Additional components on which this report is based are the authors' personal experiences in their home countries and institutions and, by extension, their personal biases. To allow authors to tap into non‐English scientific literature, those who crafted each geographical region conducted their own literature searches. As a result, no unified, systematic approach to literature searches was applied, and this may have potentially led to the omission of some relevant articles.

## CONCLUSIONS AND FUTURE CHALLENGES

The knowledge of human or animal body structures and their cellular components at the macroscopic and microscopic levels remains an essential foundation of modern human and veterinary medicine, as well as many fields of scientific investigation and the visual arts. Since the turn of the millennium, shifts in pedagogy, learning theories, technology, and changes in student demographics have dramatically altered the way the anatomical sciences are taught. Although every country and global region has individual challenges, the overall direction in which anatomy education is moving appears to be remarkably similar. Gross anatomy, histology, neuroanatomy, and embryology education have become increasingly vertically and horizontally integrated into larger curricular structures and coordinated with other basic sciences and clinical fields. Consequently, time for teaching the anatomical sciences has been reduced, mostly affecting time dedicated to laboratory sessions, and anatomical programs rely less on dissections and more on prosections, e‐learning tools, and personal electronic devices. Worldwide, academic post‐secondary institutions are shifting to the use of bodies from body donation programs for dissection‐based educational activities. The use of unclaimed, unconsented individuals is still widespread but continues to decline, sometimes resulting in a shortage of bodies for gross anatomy education. Generally, the economic wealth of individual countries influences how far anatomical sciences education has advanced in various geographic regions. Most recently, these changes were significantly accelerated by the restrictions imposed on anatomy teaching by the COVID‐19 pandemic. Overall, the challenge for anatomy educators and students in every region of the world remains to constantly adapt to an ever‐changing teaching and learning environment.

## AUTHOR CONTRIBUTIONS

**Michael Hortsch:** Conceptualization; investigation; project administration; writing – review and editing; visualization; writing – original draft. **Virginia Claudia Carneiro Girão‐Carmona:** Investigation; writing – review and editing; writing – original draft. **Ana Caroline Rocha de Melo Leite:** Investigation; writing – original draft; writing – review and editing. **Ilias P. Nikas:** Investigation; writing – original draft; writing – review and editing. **Margaret K. Gatumu:** Investigation; writing – original draft; writing – review and editing. **Nii Koney‐Kwaku Koney:** Investigation; writing – original draft; writing – review and editing. **Benjamin Arko‐Boham:** Investigation; writing – original draft; writing – review and editing. **Doris George Yohannan:** Investigation; writing – original draft; writing – review and editing. **Aswathy Maria Oommen:** Investigation; writing – original draft; writing – review and editing. **Yan Li:** Investigation; writing – original draft; writing – review and editing. **Jian Yang:** Investigation; writing – original draft; writing – review and editing. **Alexandra F. Trollope:** Investigation; writing – original draft; writing – review and editing. **Amanda J. Meyer:** Investigation; writing – original draft; writing – review and editing. **Sonya E. Van Nuland:** Investigation; writing – original draft; writing – review and editing.
